# AI-assisted histomorphological stratification of endometrial cancer: real-world validation of foundation models for molecular subtyping

**DOI:** 10.1038/s41698-026-01522-x

**Published:** 2026-06-06

**Authors:** Laura Schwarzmann, Robert Stöhr, Julius Emons, Patrik Pöschke, Katharina Seitz, Matthias Rübner, Matthias W. Beckmann, Mareike Britt Diederich, Irina Unser, Arndt Hartmann, Christian Matek, Ramona Erber

**Affiliations:** 1 Institute of Pathology, University Hospital Erlangen, Friedrich-Alexander-Universität Erlangen-Nürnberg (FAU), Comprehensive Cancer Center Erlangen-EMN (CCC ER-EMN), Erlangen, Germany; 2 Bavarian Cancer Research Center (BZKF), Erlangen, Germany; 3 Department of Gynecology and Obstetrics, University Hospital Erlangen, Friedrich-Alexander-Universität Erlangen-Nürnberg (FAU), Comprehensive Cancer Center Erlangen-EMN (CCC ER-EMN), Erlangen, Germany; 4 Institute of Pathology, University Regensburg, Comprehensive Cancer Center Ostbayern (CCCO), Regensburg, Germany

**Keywords:** Biomarkers, Cancer, Computational biology and bioinformatics, Oncology

## Abstract

Endometrial cancer (EC) is classified into four molecular subtypes with distinct prognosis and treatment implications. Despite this well-established molecular classification, morpho-molecular correlations remain understudied. Artificial intelligence (AI) enables biomarker prediction from H&E-stained whole-slide images (WSIs). However, real-world validation for EC molecular subtyping is lacking. In this study, we evaluated image-based molecular subtyping in a real-world cohort derived from routine diagnostic cases with heterogeneous image quality. We benchmarked the feature extraction with the CTransPath and UNI foundation models, demonstrating robust results across different scanner hardware and quantified performance variation by additional stain normalization. UNI-based features achieved a mean AUROC of 0.646 for *POLE*mut (*n* = 16), 0.700 for MMRd_MSI (*n* = 79), 0.684 for NSMP (*n* = 176), and 0.844 for p53abn (*n* = 18) on external real-world data. We provided human interpretations of subtype-specific morphological features. Our findings may thus lay the foundations of a more reliable framework for personalized treatment stratification based on morphologically informed molecular subtyping.

## Introduction

Traditionally, endometrial cancer (EC) was classified according to a dualistic pathogenetic model^1^ based on histopathological and clinical characteristics. While these factors remain significant, they were recently complemented by molecular data^2^. In 2013, The Cancer Genome Atlas (TCGA) published an integrated genomic, transcriptomic, and proteomic characterization of 373 EC cases, which enabled the classification into four molecular subtypes^3–5^. Their prognostic significance was confirmed and further developed for clinical routine diagnosis with the Proactive Molecular Risk Classifier for Endometrial Cancer (ProMisE) classification system. Patients with the DNA polymerase epsilon ultra-mutated subtype (*POLE*mut) exhibited an excellent prognosis, while those with an aberrant tumor protein p53 profile (p53abn) had the poorest prognosis. Patients with mismatch repair protein deficiency or microsatellite instability (MMRd_MSI) and those in the fourth subtype, characterized by no specific molecular profile (NSMP), had intermediate prognoses.

MMRd_MSI patients should be further screened for the hereditary genetic Lynch syndrome caused by an increased prevalence of 10% within this subtype^2,6^. MMRd_MSI tumors were characterized by a significantly higher tumor mutational burden than mismatch repair protein proficient or microsatellite stable tumors (MMRp_MSS), promising response to immunotherapy^7–9^. For EC, anti-PD1-based immunotherapy in combination with a multi-tyrosine-kinase inhibitor was considered for advanced, recurrent, or metastatic disease. A significant improvement was shown for MMR-deficient as well as proficient patients. Nevertheless, adverse reactions (e.g., hypothyroidism, hypertension, fatigue, diarrhea, musculoskeletal disorders) required careful evaluation of expected treatment response^10,11^. To this end, the current system is insufficient to fulfill the variable outcomes in the most prevalent subtypes, NSMP and MMRd_MSI^12^.

Furthermore, molecular subtypes were not mutually exclusive, which has been referred to as multiple-classifier cases^13^. Multiple classifier cases were reported with an incidence of 11% in a prospective cohort with 422 patients. The characteristics of multiple classifiers for *POLE*mut-p53abn were comparable to *POLE*mut, showing no recurrence, while MMRd_MSI-p53abn multiple-classifier cases differed from MMRd_MSI and p53abn regarding histotype, grade, and mismatch repair protein expression, and intermediate prognosis between the single-classified MMRd_MSI and p53abn cases for a limited number of patients. The hierarchical ProMisE algorithm did not allow for assessment of multiple molecular features in clinical routine diagnostics, with yet understudied prognostic impact^13^.

For prognostic stratification, it is crucial to combine molecular and morphological data. In previous work, morphological features of the four molecular subtypes overlapped, making it difficult for pathologists to qualify the complex association of histomorphological phenotypes with the molecular subtypes of EC^14^. Even for histological subtypes (high vs. low grade endometrioid EC, serous and other high grade EC), inter-observer variability was reported to be poor and therefore insufficient for a reliable morphology-based classification^15,16^. Deep learning (DL)-based methods have shown the ability to stably associate visual morphological features across a wide range of different cancer types with the occurrence of molecular alterations^17,18^. Compared to molecular analysis, the approach of molecular characterization based on ubiquitously available haematoxylin and eosin (H&E) stained whole slide images (WSIs) is cost-, time- and resource-effective^19,20^, thus addressing two major challenges in treatment personalization worldwide: Limited resources and reproducibility regarding cancer stratification^21^.

In the field of EC, only a few studies have addressed WSI-based prediction of the four molecular subtypes in a proof-of-concept setting^22–26^. Fremond et al.^24^ reported the first results of the four-class prediction in a large-scale study of 2028 patients from six different cohorts, from which the PORTEC-3 cohort (393 patients at high-risk, stage I-III) was used as an independent test cohort. The model im4MEC consisted of a Moco-v2 feature extractor with ResNet-50 backbone pretrained on randomly sampled tiles from each WSI of the EC cohorts and an attention-based multiple-instance learning model^27^ for the final classification. On the independent cohort slide-level predictions achieved class-wise area under the receiver operating characteristic curves (AUROCs) of 0.849 for *POLE* mutated, 0.844 for MMR deficient, 0.883 for NSMP, and 0.928 for p53 abnormal cases. Darbandsari et al.^28^ presented a new study design to stratify the biological heterogeneity of the NSMP subtype. They classified true p53abn and NSMP EC cases from two independent cohorts (290 and 614 patients) and identified a so-called p53abn-like NSMP subtype with poorer prognoses than the true NSMP cases. Recently, foundation models gained importance for feature extraction, showing superior performance for a wide range of biomarker prediction tasks. Foundation models were pretrained self-supervised on large and diverse sets of histological data designed to minimize overfitting and generalize well on data from different sources^29^. Nevertheless, benchmark studies were limited by restricted data diversity and a limited selection of clinically relevant tasks^30,31^. In the field of EC molecular subtyping, foundation models have not been used so far for studies with validation on external real-world data. In our study design, we aimed to (1) address the artificial intelligence (AI)-assisted molecular classification of a real-world EC cohort and to (2) improve performance with a foundation model-based feature extraction. Therefore, we benchmarked the foundation model UNI^32^ for generalizability on our EC cohort from clinical routine diagnostics against the well-established model CTransPath^33^, pretrained on significantly less data volume. We selected UNI, which was shown in a prior study to be a top-performing model for biomarker prediction tasks and to require relatively low computational resources, both of which predisposed it for our use case^30^.

Furthermore, in terms of clinical impact, a purely technical measurement of AI tools is insufficient. Errors of classification methods should be assessed in a combined quantitative and qualitative approach, bearing in mind their clinical implications. Explainability methods provided opportunities toward a transparent and human interpretable validation of AI-tools in computational pathology and should complement technical performance metrics (e.g., sensitivity, specificity, AUROC) to compare different tools^29,34^. As an additional analysis, we stratified the histomorphology of concordant and non-concordant predicted cases to identify subtype-specific histomorphological features as well as strengths, limitations, and potential sources of error in AI-based metrics and within the specific clinical context.

For this study, we used a fully-reproducible pipeline for feature extraction, stain normalization, and classification with a transformer-based multiple-instance learning model^18^ built upon the STAMP^35^ protocol. The classification model was fine-tuned for our use-case to predict the four molecular subtypes of EC, as well as an additional class representing non-tumor tissue, from H&E WSIs. We used the reference datasets from The Cancer Genome Atlas Uterine Corpus Endometrial Carcinoma Collection (TCGA-UCEC, finally included *n* = 454) and the Clinical Proteomic Tumor Analysis Consortium Uterine Corpus Endometrial Carcinoma Collection (CPTAC-UCEC, finally included *n* = 203) as training datasets. To ensure robust model development, we performed 10-fold cross-validation using the combined training cohorts, each with distinct data splits across training and validation sets. This iterative approach resulted in 10 pretrained models, which were subsequently tested on our independent cohort from routine clinical diagnostics at University Hospital Erlangen, characterized by a real-world, population-based profile regarding clinical characteristics and image data quality (*n* = 289 for Scanner 1 and both scanners, *n* = 287 for Scanner 2). We benchmarked the feature extraction with CTransPath^33^ and the foundation model UNI^32^, demonstrating robust results across different scanner hardware and quantified performance variation by additional stain normalization methods. We assessed morphological characteristics of the concordant and non-concordant predicted molecular subtypes with patch-level classification scores and slide-level explainability methods.

## Results

### Benchmarking of foundation models, normalization method, and scanning hardware

**Fig. 1:**
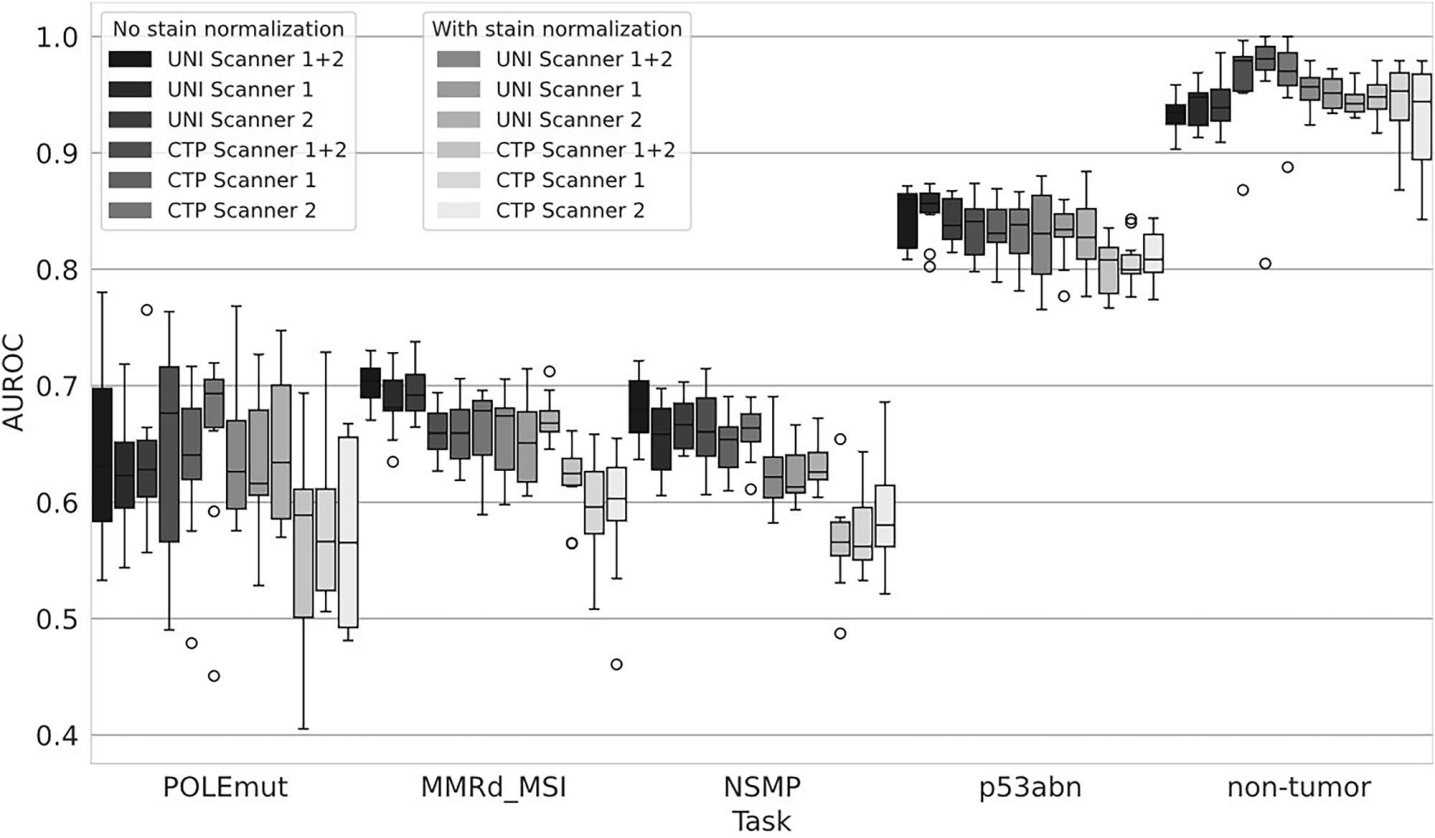
Benchmarking foundation models. Each box plot shows the mean one-vs-all class-wise area under the receiver operating characteristic curve (AUROC) for the benchmarking of UNI and CTransPath with additional stain normalization and different scanning hardware. The boxes visualize the quartiles of each distribution. The whiskers extend to the farthest data points within 1.5 times the interquartile range from the lower and upper quartiles. We performed 10-fold cross-validation to train 10 models on TCGA-UCEC and CPTAC-UCEC, which were then used for the subsequent external testing. All reported results were measured for external testing on our independent real-world cohort (*n* = 289). UNI Feature Extraction with UNI, CTP Feature Extraction with CTransPath, *POLE*mut DNA polymerase epsilon ultra-mutated subtype, MMRd_MSI mismatch repair protein deficiency or microsatellite instability, NSMP no specific molecular profile, p53abn aberrant tumor protein p53 profile, non-tumor cases with non-neoplastic tissue, normal tissue (e.g., myometrium).

We benchmarked the technical variations with the mean class-wise AUROCs. We performed 10-fold cross-validation to train 10 models on TCGA-UCEC and CPTAC-UCEC, which were then used for the subsequent external testing on the independent cohort from Erlangen (*n* = 289). Standard deviation and 95% confidence intervals of class-wise AUROCs, as well as further metrics, were reported within the supplementary results (Supplementary Tables 4 and 5). For CTransPath, the average AUROCs for raw stained images were higher than those obtained using the stain normalization method (0.753 for both scanners, 0.748 for Scanner 1 (P1000 3DHistech, Hungary), and 0.756 for Scanner 2 (S210 Hamamatsu, Japan) vs. 0.701 for both scanners, 0.700 for Scanner 1, and 0.700 for Scanner 2) (Fig. 1). Further, comparing the AUROCs class-wise, we did not observe an improvement when using Macenko’s stain normalization method. For UNI, the average AUROCs for raw stained images were also higher than those obtained with additional stain normalization (0.761 for both scanners, 0.751 for Scanner 1 and 0.755 for Scanner 2 vs. 0.688 for both scanners, 0.682 for Scanner 1 and 0.694 for Scanner 2) (Fig. 1). We did not observe an improvement for the class-wise AUROCs when using Macenko’s stain normalization method for MMRd_MSI, NSMP and p53abn, whereas we observed slightly higher AUROCs for the configuration with stain normalization for the non-tumor class with the Scanner 1 (0.940 vs. 0.951) and for the *POLE*mut class in the single scanner configuration (raw stain: 0.645 for both scanners, 0.624 for Scanner 1 and 0.633 for Scanner 2 vs. 0.639 for both scanners, 0.627 for Scanner 1, 0.645 for Scanner 2). Notably, the increased standard deviation and 95% confidence intervals for *POLE*mut indicated high variations within this subtype. The inter-scanner variability was low over all combinations (Fig. 1).

**Fig. 2:**
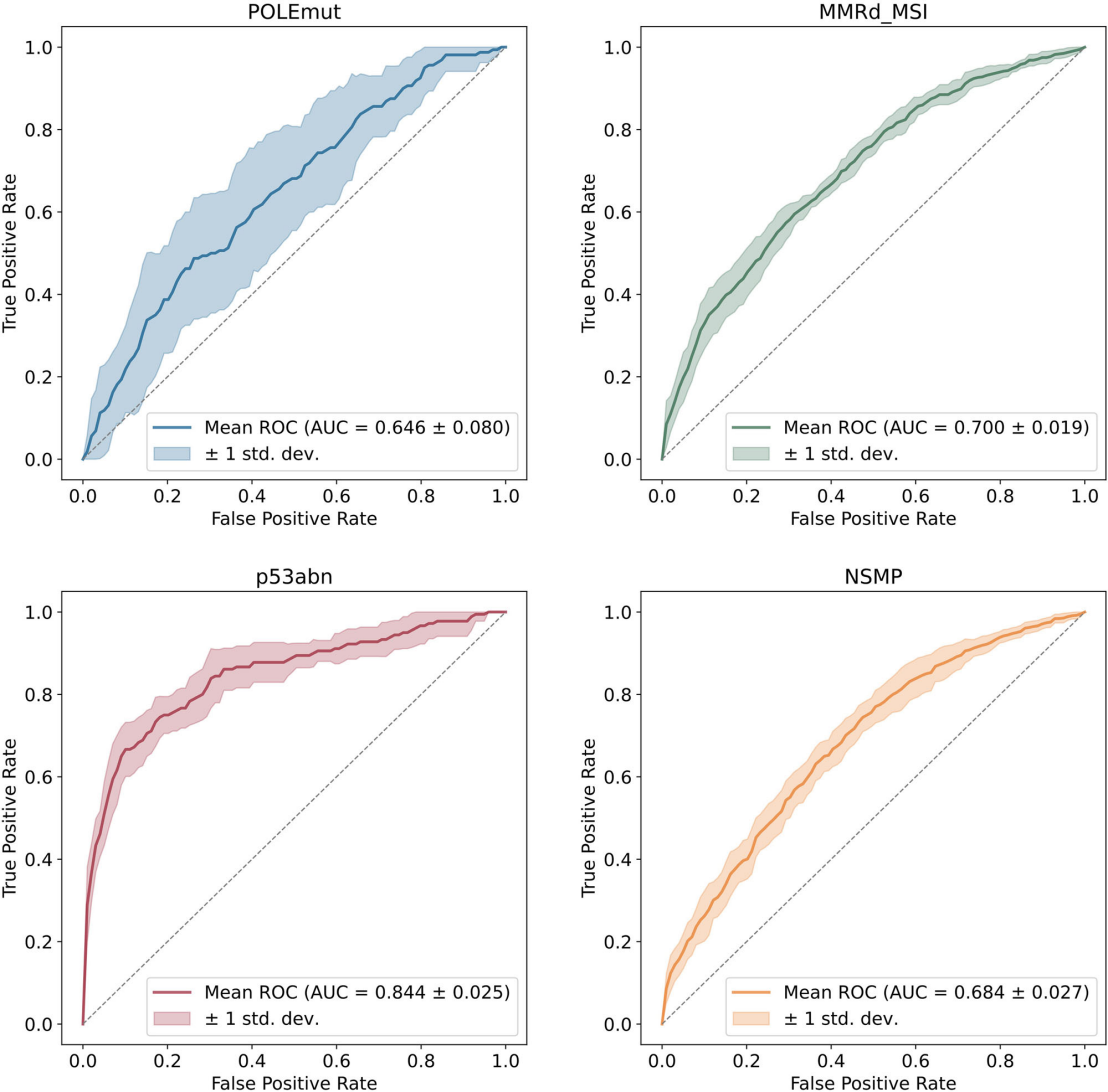
External testing after 10-fold cross-validation. We performed 10-fold cross-validation to train 10 models on TCGA-UCEC and CPTAC-UCEC, which were then used for the subsequent external testing. Mean one-vs-all class-wise area under the receiver operating characteristic curves (AUROC) were obtained from external testing on the Erlangen cohort (*n* = 289). Feature extraction was performed with UNI without stain normalization, using image data from both scanners. *POLE*mut DNA polymerase epsilon ultra-mutated subtype, MMRd_MSI mismatch repair protein deficiency or microsatellite instability, p53abn aberrant tumor protein p53 profile, NSMP no specific molecular profile.

Model performance was evaluated using mean macro-average AUROCs across the configurations (Supplementary Table 4). The combination of feature extraction with UNI, notably without the use of the stain normalization method and the use of image data from both scanners, achieved the highest mean macro-average AUROC of 0.761 for the four tumor classes and the non-tumor class on the independent test cohort. Tumor vs. non-tumor prediction is very successful (Supplementary Fig. 2). The mean one-vs-all class-wise AUROC varies from 0.646 for *POLE*mut (*n* = 16), 0.700 for MMRd_MSI (*n* = 79), 0.684 for NSMP (*n* = 176) to 0.844 for p53abn (*n* = 18), tested after 10-fold cross-validation on our independent cohort from Erlangen (Fig. 2, Supplementary Figs. 2 and 5, Supplementary Table 7). In contrast, CTransPath achieved higher mean macro-average AUROCs for the configurations with additional stain normalization.

### Evaluation of single model performance

While we presented aggregated metrics for benchmarking, the following downstream analysis required selecting a single model to generate confusion matrices and to apply explainability methods. We selected a statistically robust, top-performing single model based on validation data (Supplementary Table 6, Supplementary Figs. 3 and 4). Our model showed fair performance in external testing, achieving class-wise AUROCs of 0.766 for *POLE*mut (*n* = 16), 0.715 for MMRd_MSI (*n* = 79), 0.862 for p53abn (*n* = 18), and 0.682 for NSMP (*n* = 176) on our independent real-world test cohort. Further metrics are provided within the supplementary results (Supplementary Table 7). The confusion matrix highlighted 56.1% concordant predicted cases. Most of the predictions for MMRd_MSI, p53abn, and NSMP were concordant, while 10 of the *POLE*mut cases (*n* = 16) were predicted non-concordant. The most frequent non-concordant prediction was MMRd_MSI with 42.5% of the non-concordant predictions. Misclassified MMRd_MSI cases were predominantly predicted as NSMP (further indicated by the prefix as “morphoNSMP”). Interestingly, there was a relatively low number of morphop53abn cases (*n* = 14) among all non-concordant predictions. Note that only one case got wrongly assigned to the non-tumor class, indicating that the model confidently recognized malignancy (Supplementary Fig. 6). This sample was characterized by a small tumor size of less than 10% tumor tissue on the whole slide. We analyzed the subgroup with less than 10% tumor tissue (*n* = 48) within the supplementary data (Supplementary Fig. 9). The NSMP cases of this subgroup (*n* = 19) obtained a poor performance with an AUROC of 0.484. 13 cases were falsely classified as morpho*POLE*mut.

**Fig. 3:**
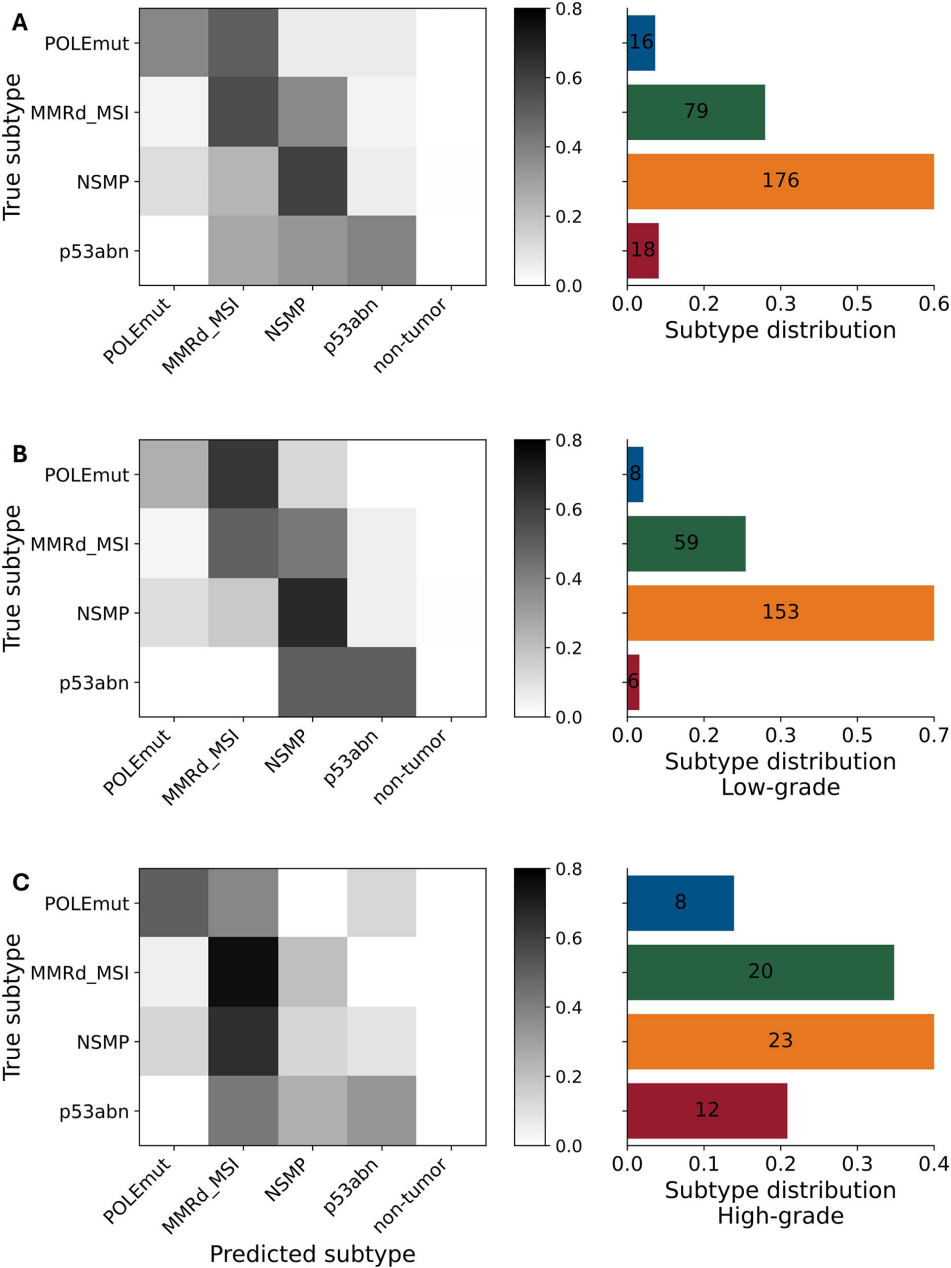
Confusion matrices. Normalized confusion matrices and distribution of molecular subtypes for the independent test cohort from Erlangen (*n* = 289, **A**), split for low-grade (*n* = 226, **B**), and high-grade subgroups (*n* = 63, **C**). All reported results were obtained from external testing on the Erlangen cohort using the top-performing single model. Feature extraction was performed with UNI without stain normalization, using image data from both scanners. *POLE*mut DNA polymerase epsilon ultra-mutated subtype, MMRd_MSI mismatch repair protein deficiency or microsatellite instability, p53abn aberrant tumor protein p53 profile, NSMP no specific molecular profile.

We stratified the results of the best single model by the histological grade of endometrioid EC (Supplementary Table 8). The majority (60.2%) of grade 1 (*n* = 107) and 2 (*n* = 119) cases, defined as the “low-grade” group^36^, were predicted concordant, while grade 3 (*n* = 63), defined as the “high-grade” group^36^, was predicted non-concordant for 58.7% of the cases (Fig. 3, Supplementary Figs. 7 and 8). The class-wise AUROC scores for the low-grade cases were 0.663 for *POLE*mut (*n* = 8), 0.721 for MMRd_MSI (*n* = 59), 0.810 for p53abn (*n* = 6), and 0.660 for NSMP (*n* = 153) on the independent test cohort. Interestingly, low-grade *POLE*mut were mainly not classified as morphoNSMP (as the other subtypes) but as morphoMMRd_MSI. The class-wise AUROC scores for the high-grade cases achieved higher AUROC scores for the *POLE*mut cases (0.861, *n* = 8) and for the p53abn cases (0.881, *n* = 12), while the prediction of the remaining subtypes MMRd_MSI (0.674, *n* = 20) and NSMP (0.590, *n* = 23) showed worse performance. The differences between the class-wise AUROC scores were not statistically significant within the unpaired *t*-test (*p* = 0.05). Confusion matrices for the low and high-grade subgroups demonstrated an association between the low-grade and the image-based prediction as morphoNSMP. The high-grade group showed an association with the prediction as morphoMMRd_MSI. We tested the association between the predictions and the grade statistically with Fisher’s exact test and accepted significance below a *p* value of 0.05. There was a significant association for the morphoNSMP (Supplementary Table 9) and the low grade, and the morphoMMRd_MSI and the high grade (Supplementary Table 10). There was no significant association between the morpho*POLE*mut or morphop53abn cases and the low- or high-grade characteristic.

**Fig. 4:**
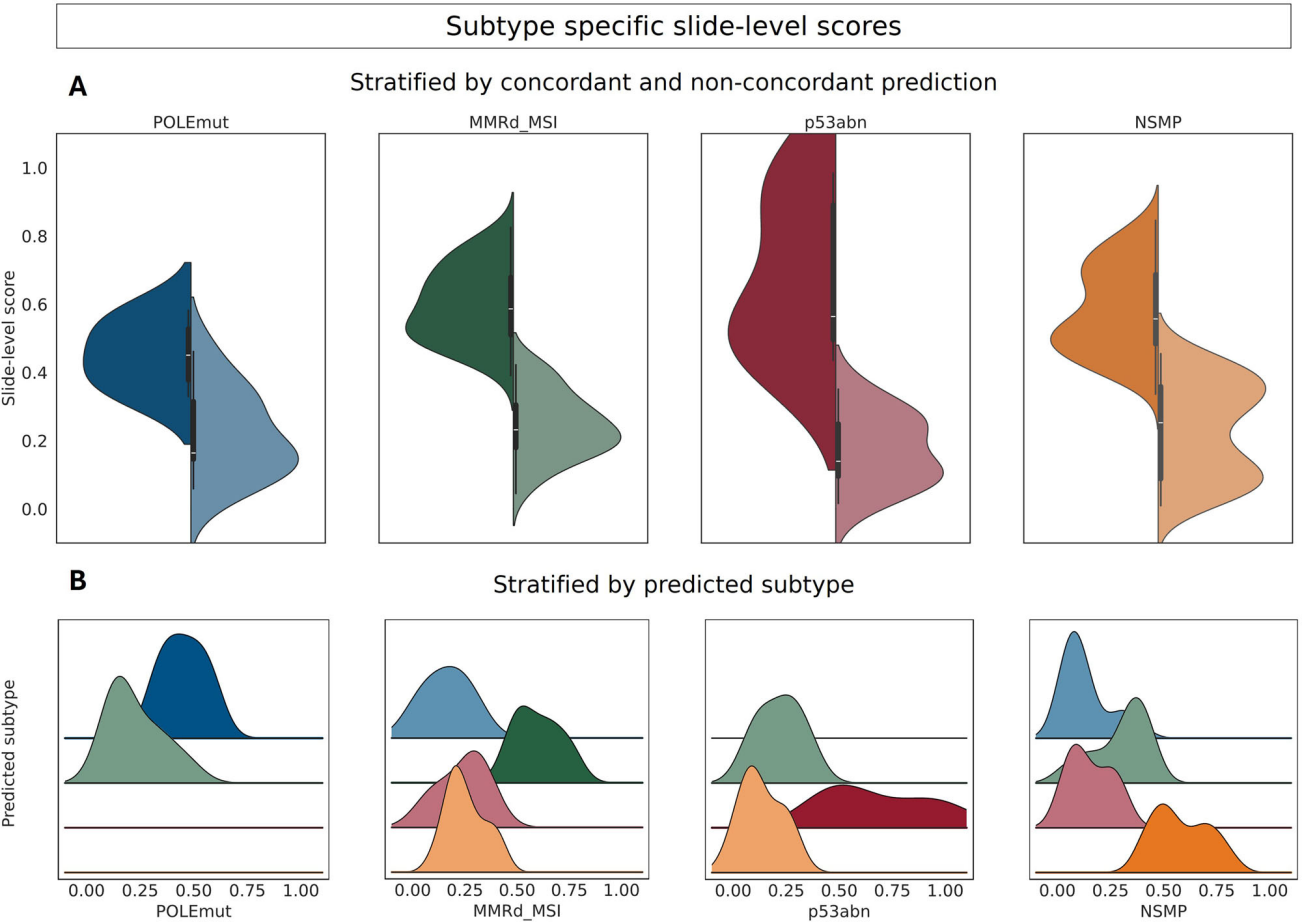
Subtype-specific slide-level scores. **A** The violin plots visualize the distribution of the slide-level scores within the true molecular subtype, stratified by concordant (left side of the violin) and non-concordant prediction (right side of the violin). The four panels correspond to the cases of one molecular subtype *POLE*mut (blue), MMRd_MSI (green), p53abn (red), and NSMP (orange). **B** The ridge plots visualize the distribution of subtype-specific slide-level scores, stratified by the predicted subtype. The colors of the ridge plots indicate the predicted subtype: *POLE*mut (blue), MMRd_MSI (green), p53abn (red), and NSMP (orange). All reported results were obtained from external testing on the Erlangen cohort (*n* = 289) using the top-performing single model. Feature extraction was performed with UNI without stain normalization, using image data from both scanners. *POLE*mut DNA polymerase epsilon ultra-mutated subtype, MMRd_MSI mismatch repair protein deficiency or microsatellite instability, p53abn aberrant tumor protein p53 profile, NSMP no specific molecular profile.

We quantified slide-level predictions to address the challenge of distinguishing overlapping morphological features. Slide-level scores presented an objective metric to measure the degree of association between the predicted label and the morphological pattern learned by the model. Figure 4 demonstrates the subtype-specific distribution of absolute slide-level scores, highlighting overlapping morphology within concordant and non-concordant predictions at a medium slide-level score. The violin for NSMP had two wider sections, which might point to density clusters for both the concordant and non-concordant predicted cases. *POLE*mut cases have lower mean and maximum slide-level scores even for concordant predictions. p53abn cases showed the highest scores for concordant predictions, while having the highest standard deviation (Supplementary Table 11). Furthermore, the ridgeplot stratified by predicted subtype demonstrated overlapping morphology for NSMP and MMRd_MSI cases at the level of 0.4. Lower slide-level scores occurred with varying non-concordant predictions, indicating increasing tumor heterogeneity.

### Morphological characteristics of the four molecular subtypes

We developed a workflow incorporating multi-resolution explainability methods. To enable comprehensive histomorphological assessment, we randomly selected representative cases covering the full range of slide-level scores. In a first step, we examined low-resolution, whole-slide heatmap overlays depicting the subtype-specific model scores (Supplementary Figs. 10–13). The heatmaps were generated using QuPath^37^, an easily applicable tool for digital slide analysis. Tumor, tumor invasive front, intra-cavitary necrosis, myometrium, and background could be identified as low or high-scored regions at low resolution. Highlighted regions could be further interpreted inside the original WSI by zooming in, facultative with filtering for specific cut-off scores. Secondly, attention maps were generated with the Grad-CAM^38^ method provided within the STAMP pipeline (Supplementary Figs. 10–13). High attention weights were correlated with specific tissue regions at a low resolution level. Altogether, high-attention regions correlated with tumor tissue, while low-attention was placed on normal myometrium, necrosis, and artifacts, which underlines our model’s ability to focus on specific histopathological features of malignancy. Finally, we analyzed specific morphological features for the highest-scoring single tiles for the molecular subtype (Supplementary Figs. 10–13). Single tile analysis was limited to the size of 224 × 224 µm, and therefore, global WSI features were unidentifiable or could be misinterpreted^29^. For example, extensive lymphocyte infiltration can be observed intratumorally or peritumorally, but the specific location is not interpretable on patches without enough contextual information from the surrounding tissue.

**Fig. 5:**
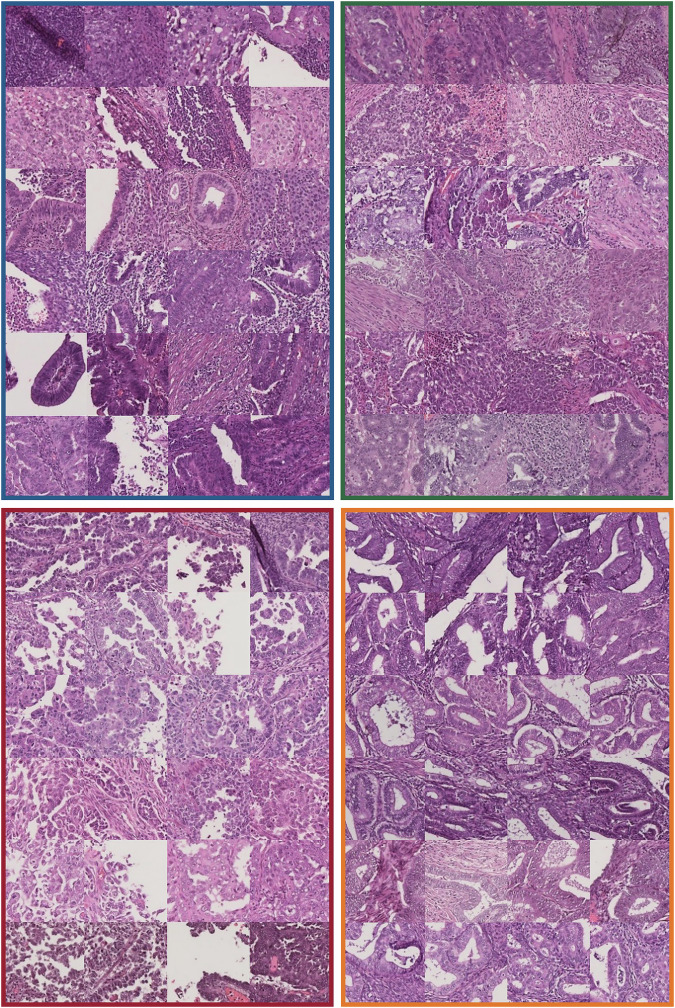
Representative selection of patches from cases with varying slide-level scores. Histomorphological analysis of six representative cases per molecular subtype predicted concordantly across varying slide-level scores. Patches in one row correspond to the same slide. Slide-level scores decrease from top to bottom row. The 100 highest-scoring patches were analyzed to screen for prototypical morphology. All slides were stained with H&E, and the patches were scanned with a Hamamatsu S210 scanner. The color of the frame corresponds to the molecular subtype: *POLE*mut DNA polymerase epsilon ultra-mutated subtype (blue, upper left), MMRd_MSI mismatch repair protein deficiency or microsatellite instability (green, upper right), p53abn aberrant tumor protein p53 profile (red, lower left), NSMP no specific molecular profile (orange, lower right).

For the selected *POLE*mut cases, concordantly predicted cases typically showed compact, partly confluent cell groups that were not fully solid but contained small abortive lumina, appearing either narrow and elongated or round with moderate to high nuclear atypia of the commonly vesicular nuclei (Table 1, Fig. 5, Supplementary Fig. 10). Lower-scoring cases tended to show glandular growth. Frequently, lymphocyte infiltration was present as dense, extensive lymphocytic infiltration across the entire patch and nested infiltration of the myometrium. Extensive infiltration was observed surrounding the tumor glands. Non-concordant cases predicted as MMRd_MSI showed luminal (so-called “dirty”) necrosis with detritus and granulocytes. Furthermore, non-concordant morpho*POLE*mut cases showed an association with highly ranked cell-rich non-tumor tissue patches (mainly endometrial stroma) that may be misinterpreted as “solid tumor tissue.”

**Table 1:** Characterization of morphological features

Morphological feature	*POLE*mut	MMRd_MSI	p53abn	NSMP
Smooth luminal border, wide lumen diameter	0	−	−	+
Microglandular	−	+	−	0
Compact growth, small abortive lumina	+	−	−	−
Solid growth	0	0	−	−
Papillary or micropapillary growth	0	−	+	−
Extensive lymphocyte infiltration	+	+	−	0
High nuclear atypia	+	0	+	−
Intraluminal necrosis	+	+	−	−

Morphological features were identified by the evaluation of highly ranked patches among a representative selection of cases with varying slide-level scores. “+” = strong morpho-molecular correlation; “0” = intermediate morpho-molecular correlation; “−” = low morpho-molecular correlation.

*POLEmut* DNA polymerase epsilon ultra-mutated subtype, *MMRd_MSI* mismatch repair protein deficiency or microsatellite instability, *p53abn* aberrant tumor protein p53 profile, *NSMP* no specific molecular profile.

Concordantly predicted MMRd_MSI cases showed mainly small tubules (microglandular or “microacinar-like”) growth with partly confluent features, but no true solid growth (Table 1, Fig. 5, Supplementary Fig. 11). Frequently, patches corresponded to the myometrial invasion front and showed intramyometrial features of small, non-gland-forming cancer cell clusters. Lymphocyte infiltration was moderate to high among all selected cases in the sense of an extensive infiltration as well as densely scattered cells between myometrial muscle cells and tumor glands, which was also observed for the non-concordant morphoMMRd_MSI patches. Intraluminal necrosis with granulocytes and intraluminal mucin and secretion were frequently observed among molMMRd_MSI and morphoMMRd_MSI patches. Cases with lower slide-level scores tended to show less complex glandular growth. Glands were mainly small and round with a relatively small intraluminal space.

Concordant p53abn cases showed mainly serous-like features: Papillary to micropapillary growth with sharply thickened nuclear membrane and high nuclear atypia (Table 1, Fig. 5, Supplementary Fig. 12). Poor fixation may have mimicked serous-like features on some patches. Lymphocyte infiltration was low among the majority of selected cases. Interestingly, p53abn cases with low slide-level scores and morphop53abn cases showed patches with background (e.g., glass) among their highest scored patches. The attention maps indicated high attention at background regions without tissue, where glass reflections were misinterpreted by the model.

The histomorphological analysis of NSMP cases highlighted glandular growth with medium-sized tubular glands with smooth luminal borders and back-to-back formation as a substantial pattern among most selected slide-level scores (Table 1, Fig. 5, Supplementary Fig. 13). Other cases showed cribriform-like tumor cell groups or secretory transformation. The analyzed concordantly predicted cases showed low lymphocyte infiltration and low nuclear atypia. Non-concordant predictions showed increasing intraluminal mucin and necrosis, moderate to high lymphocyte infiltration, decreasing back-to-back arrangement, and confluent glandular growth.

## Discussion

In this work, we used state-of-the-art foundation models to develop a classifier for EC molecular subtyping based on H&E WSIs. Overall, we achieved fair performance on a real-world, population-based test cohort from routine clinical diagnostics. In our use-case, the UNI foundation model achieved the highest mean macro-average AUROC of 0.718 for the four tumor classes with model configuration no stain normalization and use of both scanners, outperforming CTransPath with a mean macro-average AUROC of 0.701 for the four tumor classes with the same model configuration. This finding suggests superior generalizability of foundation models. Our models demonstrated robust results across two different scanner types, even without applying stain normalization, suggesting a subordinate role of varying scanner hardware for the final prediction. This is a considerable and previously unreported insight regarding multi-institutional validation. Overall, the performance of UNI and CTransPath among the different configurations was similar. From a practical point of view, the omission of stain normalization may offer advantages in resource-constrained settings.

In comparison with previous work, our model achieved moderate one-vs-all classification performance as measured by the AUROC. We achieved inferior class-wise AUROCs compared to the validation for the PORTEC-3 high-risk patients reported by Fremond et al.^24^ (AUROC of 0.849 for *POLE* mutated, 0.844 for MMR deficient, 0.883 for NSMP, and 0.928 for p53 abnormal cases). The PORTEC-3 cohort was collected and pre-selected within the context of a clinical trial and includes high-risk patients only, as well as non-endometrioid EC, limiting transferability to clinical routine diagnostic settings. Differences in cohort composition might cause a higher AUROC for p53abn cases of the PORTEC-3 cohort than in our work (0.928 vs. 0.862). Note that the model im4MEC was pretrained on approximately twice as many patients (*n* = 1633) as in our study design (*n* = 657, incl. non-tumor slides). In contrast, our real-world cohort was selected consecutively and characterized by a heterogeneous and wide spectrum of risk, comorbidities, and therapy adherence. Due to differences in cohort composition, data availability, and training procedures, a direct comparison to im4MEC was not feasible, limiting cross-study comparability. Consequently, our reported performance should be interpreted as relative performance within a controlled experimental framework, providing a real-world evaluation. Recently, Wagner et al.^39^ presented a preprint benchmarking a real-world patient cohort (*n* = 720) with foundation models, achieving AUROC up to 0.78 for the EC subtyping. Some detected histomorphological features were comparable with our work, in particular, high inflammation for *POLE*mut cases, moderate inflammation for MMRd_MSI, and subtype-specific patterns in growth architecture, which supports the general presence of AI-detectable features within real-world cohorts. However, Wagner et al. reported a predominance of morpho*POLE*mut within the non-concordant predictions, findings that are inconsistent with Fremond et al. and our results.

The four molecular subtypes of EC are characterized by different clinical and pathological characteristics. In the following, we discussed our AI-assisted histomorphological stratification in the subtype-specific context. *POLE*mut EC patients typically present at a relatively young age, with lower BMI, and early FIGO stage. Despite clinicopathological high grade, they have an excellent prognosis^3,40,41^. No adjuvant treatment regimen is needed for stage I and II^2,42^. Recent evidence supported de-escalated regimens even for high-risk patients^43,44^. An AI-assisted morphological stratification for this subtype of EC may be clinically beneficial as a pre-screening tool to reduce the time-consuming and costly polymerase chain reaction (PCR) tests for the ultra-mutated subtype of DNA polymerase epsilon. The morphology of *POLE*mut cases has been characterized by a higher prevalence of peritumoral lymphocytes (Crohn’s-like infiltrate) and tumor-infiltrating lymphocytes (TILs), scattered tumor giant cells, and a broad front invasion type in previous studies^14,45^. Additionally, we identified extensive lymphocyte infiltration as a relevant pattern in highly ranked *POLE*mut patches among all slide-level scores. The association of *POLE*mut and MMRd_MSI features was also observed in previous work of AI-based EC classification reported by Fremond et al.^24^, as is apparent in the high proportion of non-concordant predictions classified as morphoMMRd_MSI. In our work, *POLE*mut showed an association with highly ranked non-neoplastic, stromal cell-rich tissue, which may be misinterpreted as “solid tumor”, indicating a limitation for morphology-based approaches, especially for slides with a small proportion of tumor tissue.

Among MMRd_MSI EC patients, intermediate and advanced stages are more prevalent. High tumor grades and endometrioid histological subtype are frequent^40,41^. Prognosis is intermediate and significantly worse compared to *POLE*mut cases^3^, while histologically, overlapping features (e.g., substantial lymphovascular space invasion, high grade) have been reported. The prevalence of peritumoral lymphocytes and TILs is moderate compared to *POLE*mut cases but higher than for the remaining subtypes^14^. While *POLE*mut cases tended to be misclassified as morphoMMRd_MSI, we could not observe this association for molMMRd_MSI cases. MMRd_MSI cases were mainly predicted as non-concordant with NSMP. We found moderate to high lymphocyte infiltration among highly ranked patches for MMRd_MSI.

p53abn EC is characterized by aggressive biology resulting in poor prognosis^3^. Patients typically present at an older age and with a lower BMI. Non-endometrioid morphology, advanced stage, and high grade are more frequent^40,41^. The prognostic implication of histological type and low or high grade was unconfirmed for this molecular subtype^46,47^. p53abn EC frequently presented serous-like histology with papillary, micropapillary, or glandular architecture with high nuclear atypia and increased mitotic activity. TILs are less frequently reported^14,48,49^. In our study, the test cohort was limited to the endometrioid subtype of EC, while the training cohort included cases of serous and mixed carcinoma as well. Nonetheless, the p53abn group achieved the highest AUROCs, indicating strong morpho-molecular correlations for endometrioid p53abn EC. In our work, highly ranked patches showed mainly serous-like features and low lymphocyte infiltration. The high frequency of serous-like features may be biased by the predominance of p53abn molecular alteration among the serous and mixed carcinoma cases in the training dataset. Models pretrained only on endometrioid EC may be interesting, regarding morphological features exclusive to endometrioid p53abn EC. Previous studies with higher proportions of serous or other non-endometrioid high-grade p53abn carcinoma cases tended to achieve higher performance metrics for this subtype^24,39^. More distinct morphological features between the molecular subtypes—and consequently higher performance metrics—are expected due to differences in histological subtypes.

The remaining cases with NSMP make up the majority of EC. Clinical characteristics are associated with intermediate age and increased BMI, frequently presented at low stage^3,40,41^. In contrast to other molecular subtypes, NSMP cases are heavily affected by tumor grade and histotype^47^. The NSMP group is heterogeneous and showed a wide range of clinical outcomes and tumor biology^3^. Recently, increased immunohistochemical (IHC) L1CAM expression, *CTNNB1* and *ARID1A* mutations, and progesterone receptor negativity were associated with an inferior prognosis^50–53^. A predominant glandular growth with smooth luminal borders and a relatively low presence of lymphocytes appeared frequently among our selected highly ranked patches for NSMP. Both features were previously reported to correlate with NSMP and were mentioned analogously in the AI-based work of Fremond et al.^14,24,53^. Darbandsari et al. stratified NSMP cases based on histopathological images and revealed a subgroup of “p53abn-like NSMP” with statistically significant inferior clinical outcome and morphologically increased nuclear atypia. In our work, 10 of 176 NSMP cases were classified as morphop53abn. The morphop53abn case of our histomorphological analysis also showed high nuclear atypia among the highly ranked patches. Regarding prognostic stratification, the impact of classification scores for the NSMP subtype should be further investigated for the correlation with distinct clinical outcomes within this biologically heterogeneous group.

Our detailed assessment of distinct and overlapping morphological features within the true and the predicted molecular subtype showed that relative slide-level and patch-level scores are a key factor for the AI-assisted histomorphological stratification of EC. Absolute slide-level labels and single technical performance metrics are not sufficient to capture the heterogeneity of EC tumor biology. We reported distinct and overlapping morphological features within the true and predicted molecular subtypes, highlighting subtype-specific histomorphology differing in frequency of occurrence and degree of expression across different slide-level scores. Specific features for tumor architecture (e.g., growth pattern, nuclei atypia) and immune cell infiltration were observed on independent highly ranked patches. Artifacts on patches, such as glass reflection for p53abn and tissue with poor fixation, can lead to misinterpretation by the model and are more frequent among cases with lower slide-level scores or non-concordant predictions. Both artifact patterns may be addressed by non-WSI-based approaches. Classification models for pre-selected regions of interest, either human or AI-assisted, may facilitate distinguishing the predictive value of patterns in tumor architecture or immune cell infiltration and avoid misclassifications caused by artifacts. Moreover, exclusion of non-relevant tissue may lead to a significant reduction in image data volume, hence alleviating the resource demands of the software systems involved, which may be particularly critical in a resource-constrained clinical setting. Furthermore, from a clinical point of view, molecular classification of tissue specimens obtained from curettage material or endometrial biopsies is equivalent to the molecular diagnosis on the hysterectomy specimen, with only a few exceptions, and must be performed for fertility-sparing treatment^2,42^. AI-based molecular subtype classification remains to be investigated for curettage and biopsies in EC, which represent a highly relevant scenario.

While providing a comprehensive evaluation of the potential of AI-based molecular classification in EC, the present study has limitations. As far as the training data included is concerned, both the overall number of patients and their clinical heterogeneity were limited to the two publicly available training datasets from TCGA and CPTAC^54^. Similarly, the test cohort is limited to one, single institutional cohort with a relatively low number of cases, especially for the *POLE*mut and p53abn subtypes. The relative scarcity of *POLE*mut and p53abn cases complicated the morphology analysis due to low numbers of non-concordant and concordant predicted cases. In particular, the mean AUROC scores for *POLE*mut cases showed unstable performance of 0.646 and an increased standard deviation of 0.080 (model configuration UNI, no stain normalization, both scanners). The lower proportion of *POLE*mut and p53abn among endometrioid EC is a biological limitation and was consistently reported in previous work^3,4,43,55^. However, foundation models are considered promising for robust performance on tasks with class imbalance, particularly in the case of biologically rare tumor subtypes such as the *POLE*mut class in EC. Accordingly, our real-world validation represents a particularly challenging yet highly relevant use-case for real-world evaluation of foundation models. The differentiation of technically challenging small sample sizes and the presence of sufficiently distinctive histomorphological features should be investigated further to assess improvements in predictive performance of the *POLE*mut subtype. We therefore consider our findings as a real-world performance estimate under constrained conditions, rather than a definitive assessment of subtype classification accuracy. Our test cohort included endometrioid EC only, restricting the morphological analysis to this histological subtype. Indeed, the histological subtype was suggested to be prognostically insignificant within all molecular subtypes except NSMP^47^. The use of a training cohort restricted to endometrioid histological subtype would be very interesting from a pathologist’s perspective. The comparison of endometrioid against mixed cohorts for the use-case of EC should be further investigated within a robust study design and sufficient sample sizes, allowing a detailed comparison of histomorphological characteristics. Moreover, analysis of morphological features on a patch level is limited to a size of 224 × 224 µm without context information on the surrounding tissue architecture. The number of selected cases for morphological interpretation was limited to six concordantly predicted cases and one non-concordant case for all occurring combinations. The correlation of AI-based prediction and clinical endpoints represents a key step toward clinical translation. Currently, we cannot fulfill correlative analysis to assess clinical impact within the scope of this paper, focusing on technical and histomorphological analyses. At the same time, our real-world performance estimation is positioned as a necessary intermediate step in the translational pipeline. The integration of clinical endpoints, as well as the evaluation of clinically meaningful decision frameworks (e.g., one-vs-all or one-vs-one classification strategies) should be further addressed as the clinical impact of the four molecular subtypes of EC for prognostic stratification is well established^3–5^ supporting the potential correlation between AI-based predictions and clinical endpoints.

Another critical step in the clinical translation of AI-assisted molecular classification of EC is reliable real-world validation. The concept of reproducibility and reusability of DL algorithms in computational pathology has been addressed only passingly in previous work. Ideally, both code and data should be available to reproduce findings and generate prospective evidence^19,56^, a situation which is often hampered by data protection aspects, especially for real-world cohorts. Research initiatives like TCGA and CPTAC provide large-scale publicly available datasets for EC to set up standardized DL-models^21^. Using this data for model pretraining enables a reproducible benchmark for future validation studies. Nonetheless, a critical preparation of a publicly available dataset is needed to avoid model training on slides without tumor content or very poor slide quality. Within this work, we propose a framework that clearly defines exclusion criteria for the slide quality assessment step. We exclude a non-negligible number of cases for predefined criteria and encourage researchers to critically evaluate their datasets. Our evaluation of the TCGA and CPTAC cases is available via the supplementary data. To ensure reliability, our workflow is fully-reproducible with the STAMP protocol and pretraining restricted to publicly available datasets, inviting pathologists to prospectively validate AI-assisted morphology analysis in EC with real-world data.

## Methods

### Data

H&E WSI, molecular, and clinicopathological data were obtained from 1115 patients diagnosed with EC, sourced from three independent cohorts: The Cancer Genome Atlas Uterine Corpus Endometrial Carcinoma Collection (TCGA-UCEC, *n* = 529), the Clinical Proteomic Tumor Analysis Consortium Uterine Corpus Endometrial Carcinoma Collection (CPTAC-UCEC, *n* = 254), and our real-world cohort from routine clinical diagnostics at University Hospital Erlangen (*n* = 332) (Supplementary Fig. 1). After evaluating the available slides, molecular labels, and tissue quality, 657 patients were included in the training cohort, while 286 patients were assigned to the test cohort.

#### Data sources and availability

For the data sources and availability of the TCGA-UCEC and the CPTAC-UCEC cohort, we refer to the data availability part. Both cohorts’ image and clinical data are publicly available.

**Fig. 6:**
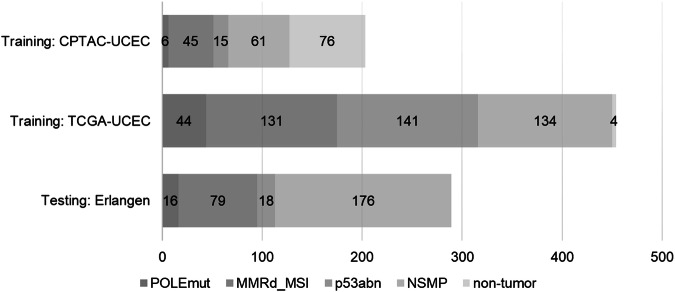
Cohort distribution. Molecular stratification of included cases of the training cohorts CPTAC-UCEC and TCGA-UCEC and the independent test cohort from Erlangen. TCGA-UCEC The Cancer Genome Atlas Uterine Corpus Endometrial Carcinoma Collection, CPTAC-UCEC Clinical Proteomic Tumor Analysis Consortium Uterine Corpus Endometrial Carcinoma Collection, *POLE*mut DNA polymerase epsilon ultra-mutated subtype, MMRd_MSI mismatch repair protein deficiency or microsatellite instability, p53abn aberrant tumor protein p53 profile, NSMP no specific molecular profile, non-tumor cases with non-neoplastic tissue, normal tissue (e.g., myometrium).

The study protocol conformed to the principles of the Declaration of Helsinki. The samples from Erlangen were collected retrospectively from the institute’s archive. The samples from Erlangen were collected retrospectively from the institute’s archive. The admission to the study has been approved by the Ethics Committee of the Friedrich-Alexander University Erlangen-Nürnberg (Nr. 252_20B), allowing the use of de-identified data for research without the requirement for individual patient consent. The requirement for written informed consent was waived because all data were de-identified. After review and quality control, 332 female patients with endometrioid EC primarily diagnosed between 2008 and 2019 were included (Fig. 6). For all cases, the most representative WSI was selected by a board-certified pathologist experienced in gynaecopathology (R.E.). Slides from the Erlangen cohort were digitized at 40-fold magnification using as “Scanner 1” a P1000 scanner (3DHistech, Hungary) and as “Scanner 2” a S210 scanner (Hamamatsu, Japan). For all patients from the Erlangen cohort, molecular classification had been obtained in-house as detailed below.

While the two publicly available datasets used in this work were collected in a study context, the Erlangen dataset was compiled solely using data from routine diagnostic pathology as used at University Hospital Erlangen. In that context, we assessed AI-based biomarker prediction in a real-world setting with regard to patients’ characteristics and slide quality.

#### Image data

All analyzed histological image data were generated from formalin-fixed paraffin-embedded tumor specimens from EC of various grades, stages, and histological subtypes (e.g., endometrioid, serous, mixed endometrioid and serous), including hysterectomy specimens and biopsies. Tissue sections were H&E-stained and contained a tumor percentage of 5–100% with a minimum tumor area of 3 mm^2^. All slides were digitized at 20× or 40× objective magnification and saved as WSI in the respective vendor-specific format. The preprocessing software downscales all slides to a resolution of 1.14 µm/px, which allows the usage of different magnification levels that are higher than 1.14 µm/px. Exclusion criteria were defined as no available slide or molecular label or criteria of very poor slide quality (tumor size smaller than 3 mm^2^ or over 50% of tumor area with necrosis, thick cut, and blurriness) (Supplementary Table 1). The case-specific exclusion criteria for TCGA-UCEC and CPTAC-UCEC are available via the supplementary data file.

#### Molecular and clinicopathological data

Molecular classes of EC were defined corresponding to the ProMisE Classification^4^. For all samples of the Erlangen cohort, the molecular characterization was made in-house. IHC was performed for detecting the presence or absence of one or more of the mismatch repair proteins to find cases corresponding to the “MMRd_MSI” label, sequencing for mutations in the DNA polymerase-ɛ (*POLE*) exonuclease domain in exons 9–14 (*POLE*mut), which corresponds to the ultra-mutated subtype, and an aberrant IHC profile for tumor protein 53 (p53abn) analogous to the copy number high subtypes of the TCGA cohort. The remaining, p53 wild-type cases were defined as having NSMP analogous to the copy number low subtypes of the TCGA cohort.

We included 577 patients with tumor tissue and 80 patients with non-tumor tissue for the training cohort and 289 patients with tumor tissue for the test cohort (Fig. 6, Supplementary Table 2). The histological subtypes in the TCGA-UCEC cohort are heterogeneous: 19.7% are serous carcinoma and 4.2% are mixed serous and endometrioid carcinoma, while 76.0% are endometrioid carcinoma. All cases of the CPTAC-UCEC cohort are endometrioid carcinoma. The test cohort from Erlangen is composed of 100% endometrioid carcinoma (Supplementary Table 3). The most frequent subtype among all cohorts is NSMP, while the *POLE*mut cases are rare among all cohorts. This frequency distribution corresponds to the prevalence of the four molecular subtypes previously reported for EC^4,12^. The representation of the biological variability is limited by the absolute number of 16 *POLE*mut cases and 18 p53abn cases in the test cohort. Managing class imbalance is a challenge for the model, arising from the natural, real-world distribution of EC subtypes.

### Model pipeline

For developing a digital algorithm to predict the molecular subtype from digital H&E image data, we used STAMP, a modular, open-source framework developed by El Nahhas et al.^35^. The pipeline supports essential principles for the implementation of DL algorithm on routine diagnostic data: Using foundation models trained on large and diverse sets of histological data, the method is designed to minimize overfitting and generalize well on data from different sources^31^.

#### Data splitting

Training and validation data are used for modeling (by default, 80% of the whole dataset; 64% training and 16% validation). The test data is not used at training time, but is kept for evaluation of the classification model, either with unseen data of the same cohort or with data from an external dataset (by default, 20% of the dataset). Differences in technical and biological variability of histopathological image data can cause batch effects^54^. To address this bias, we repeated 10 iterations of modeling with the whole dataset of the two training cohorts with 10 different data splits across training and validation. The random data split was performed using scikit-learn’s random_state argument, ensuring reproducibility across all different configurations. Finally, the availability of the cohort from Erlangen as an external dataset made it possible to evaluate the model’s generalizability on the 10 pretrained, cross-validated models.

#### Image preprocessing

WSIs are characterized by typical data sizes of several gigabytes. For downstream processing using typical capacities, each WSI is divided into N smaller image data units called patches of 256 µm (e.g., an RGB image of size 224 × 224 pixels). This step reduces memory requirements and furthermore enables parallelized model structures^19^. Patches with blurry image content or white-space background, defined as two-edged tissue, were detected by a Canny edge detector and subsequently excluded^57^. No further patches or image regions of the WSI were excluded. Subsequently, feature extraction was performed on each of N patches per WSI. H&E-stain deviations are normalized to a reference stain intensity by using the Macenko stain normalization technique^58^. No additional data augmentation is performed during preprocessing, but an augmentation is integrated in the self-supervised learning-based feature extraction with CTransPath^33^.

#### Feature extraction

We use two different state-of-the-art feature extractors with comparable transformer-based architecture: CTransPath^33^ and the model UNI^32^. Self-supervised pretraining on different compositions and sizes of histopathological data expects performance variations in our use case (e.g., 32,000 WSIs for CTransPath and more than 100,000 WSIs for UNI). In general, feature extraction results in a vector of encoder-specific dimensionality (e.g., 768 for CTransPath and 1024 for UNI) *M* for *N* tiles per slide representing image information in computer-processable data with dimension *N* × *M*. For patients with more than one WSI feature matrices of these WSIs were concatenated after feature extraction.

#### Classification model

The classification in slide-level prediction scores for the four molecular subtypes and the non-tumor class was performed with a transformer-based multiple instance learning model provided within the STAMP protocol^35^. The Vision Transformer model has two layers with eight attention heads. We used an early-stopping callback, a batch size of 64, and a bag size of 512. We used hyperparameter tuning to improve the model’s results for our specific use case. The standard configuration file of the STAMP source code does not allow changing hyperparameters, so we changed hyperparameters (e.g., the mlp dimension = 512, learning rate = 1e-3 for an Adam optimizer, weight decay = 1e-4, drop out = 0.25) as well as some additional minor changes for further statistical and visualization analyses in our local version of the cloned conda environment. We performed 10-fold cross-validation with the cohorts TCGA-UCEC and CPTAC-UCEC. This was repeated for all configurations of feature extractors, scanning hardware, and the use of stain normalization. These models were used for the deployment and evaluation on the external cohort from Erlangen. The random data split was performed using scikit-learn’s random_state argument, ensuring reproducibility across all different configurations. To ensure balanced proportions of the tumor subtypes and the non-tumor cases among the 10 data splits, we used a stratified cross-validation. No cases were excluded during model training. Consequently, the overall class distribution remained unbalanced, which represented an acceptable constraint for the model.

#### Explainability of visual interpretation

Heatmap overlays over the H&E tissue morphology were used to visualize attention distribution and assess their morphologic relevance. Attention maps were generated with the Grad-CAM^38^ method provided within the STAMP pipeline. Additionally, we developed a visualization of patch-level values as a QuPath^37^ implementation as an easy-to-applicable tool for pathologists’ digital diagnostic workflow. Highlighted regions could be further interpreted inside the original, high-resolution WSI by zooming in, facultative with filtering for specific cut-off scores. Low-resolution WSI-overlay maps are provided via the supplementary data for representative cases. Heatmaps and the selection of highly ranked tissue patches are essential for our analyses of subtype-specific morphology. The interpretation of visualization methods of neural networks is limited by their highly complex, non-linear aggregation processing. The non-linear connection between the highest-scoring tiles and the final slide-level prediction is a bias for the interpretation of morpho-molecular associations.

### Statistical analysis

The performance of the models was measured using the class-wise and macro-average AUROC for the deployment on the independent cohort from Erlangen. For benchmarking foundation models, the normalization method and scanning hardware mean AUROC scores from the 10 models deployed on the independent test data were used for statistical and graphical evaluations. The predictions were made per patient; for patients with more than one available WSI, the corresponding feature matrices were concatenated for the final patient-level prediction. In the supplementary results, we also calculated the area under the precision-recall curve for the top-performing configuration, which was further evaluated. Statistically significant association between the class-wise predictions and low and high grade group were calculated with Fisher’s exact test (*p* = 0.05).

## Supplementary information

Supplementary Information

Supplementary Data 1

## Data Availability

Slides for the TCGA-UCEC cohort are available from the Genomic Data Commons (GDC) Data Portal: https://portal.gdc.cancer.gov/projects/TCGA-UCEC. The molecular labels are available from cBioPortal: https://www.cbioportal.org/study/summary?id=ucec_tcga_pan_can_atlas_2018. Slides for CPTAC-UCEC cases are available from the Cancer Imaging archive: https://www.cancerimagingarchive.net/collection/cptac-ucec/. The molecular labels for the cases used in this study are according to a previous work by Dou, Y. et al.^59^. The final included cases of the TCGA-UCEC and CPTAC-UCEC cohorts are available from our supplementary data 1 file. As the Erlangen EC cohort is derived from direct routine practice, it cannot be published as a whole for reasons of data privacy. However, the authors will endeavor to provide additional cohort information upon reasonable request. The study protocol conformed to the principles of the Declaration of Helsinki. The samples from Erlangen were collected retrospectively from the institute’s archive. The admission to the study has been approved by the Ethics Committee of the Friedrich-Alexander University Erlangen-Nürnberg (Nr. 252_20B), allowing the use of de-identified data for research without the requirement for individual patient consent. The requirement for written informed consent was waived because all data were de-identified.

## Code availability

We used the open-source STAMP software (Version 1.1.0) for the benchmarking experiments. The source code is available via GitHub (https://github.com/KatherLab/STAMP/tree/v1).
