# Supplementary material for: AI-assisted histomorphological stratification of endometrial cancer: real-world validation of foundation models for molecular subtyping
Source: NPJ Precis Oncol. 2026 Jun 6;10:205. doi: 10.1038/s41698-026-01522-x (PMC13242518; doi:10.1038/s41698-026-01522-x)
Supplement: Supplementary file 1 — Supplementary Information [file 41698_2026_1522_MOESM1_ESM.pdf]

Supplementary Data

Supplementary Materials

Cohort Characterisation

Patient collection for the Erlangen cohort

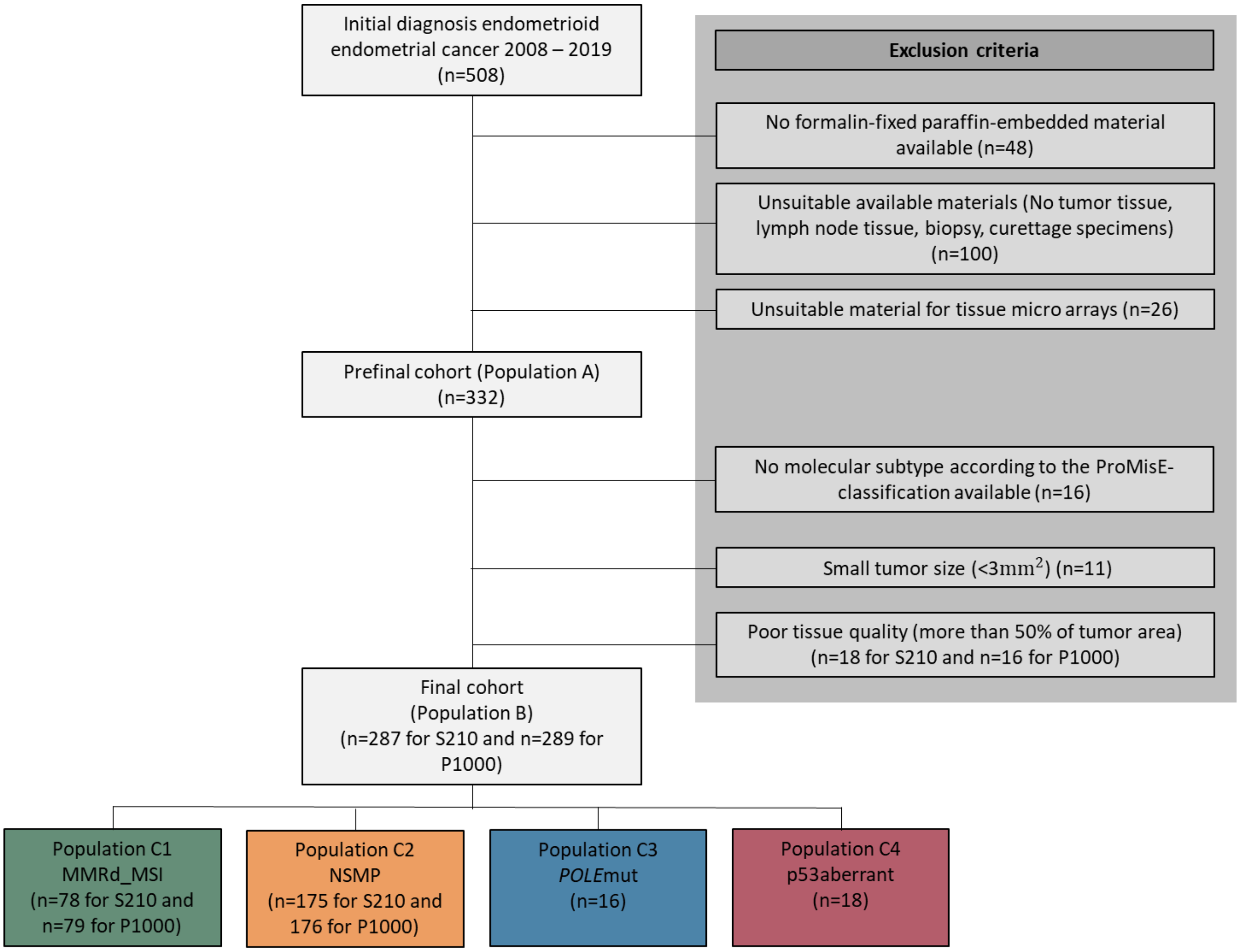

Supplementary Figure 1: Flowchart. The flowchart shows the final cohort from Erlangen and the number of excluded cases sorted by exclusion criteria.

Exclusion criteria

**Exclusion criteria**	**EC_Erlangen Scanner 1**	**EC_Erlangen Scanner 2**	**CPTAC-UCEC**	**TCGA-UCEC**
Number of patients absolute	332	332	254	529
Number of patients relative (%)	29,8	29,8	22,8	47,4
Exclusion No slide available	0	0	0	42
Exclusion No molecular label available	16	16	119	22
Exclusion tumor size <3mm2	11	11		4
Exclusion poor tissue quality non specific			8	15
Exclusion >50% necrosis in tumor area	13	13		
Exclusion >50% thick cut (with bluriness)	2	2		
Exclusion >50% bluriness in tumor area	1	3		
Final included for feature extraction tumor	289	287	127	450
Final included for feature extraction non-tumor			76	4

Supplementary Table 1: Criteria for excluded cases. The table shows the number and criteria for excluded cases sorted by cohorts. EC_Erlangen Scanner 1 = WSIs scanned by scanner P1000, EC_Erlangen Scanner 2 = whole slide images (WSI) scanned by scanner S210, TCGA-UCEC = The Cancer Genome Atlas Uterine Corpus Endometrial Carcinoma Collection, CPTAC-UCEC = Clinical Proteomic Tumor Analysis Consortium Uterine Corpus Endometrial Carcinoma Collection.

Supplementary Data 1: Case selection TCGA-UCEC and CPTAC-UCEC. The final included cases and the criteria for excluded cases of the TCGA-UCEC and CPTAC-UCEC cohorts are available from our supplementary data 1 file.

Molecular characteristics

**Molecular subtype**	**EC_Erlangen Scanner 1**	**EC_Erlangen Scanner 2**	**CPTAC-UCEC**	**TCGA-UCEC**
All included	289	287	127	450
*POLE*mut	16	16	6	44
(%)	5,54	5,58	4,72	9,78
MMRd_MSI	79	78	45	131
(%)	27,34	27,18	35,43	29,11
p53abn/CN high	18	18	15	141
(%)	6,23	6,27	11,81	31,33
NSMP/CN low	176	175	61	134
(%)	60,90	60,98	48,03	29,78

Supplementary Table 2: Molecular stratification. The table shows the molecular stratification of included cases with tumor tissue sorted by cohorts. EC_Erlangen Scanner 1 = WSIs scanned by scanner P1000, EC_Erlangen Scanner 2 = whole slide images (WSI) scanned by scanner S210, TCGA-UCEC = The Cancer Genome Atlas Uterine Corpus Endometrial Carcinoma Collection, CPTAC-UCEC = Clinical Proteomic Tumor Analysis Consortium Uterine Corpus Endometrial Carcinoma Collection, *POLE*mut = DNA polymerase epsilon ultra-mutated subtype, MMRd_MSI = mismatch repair protein deficiency or microsatellite instability, p53abn/CN high = aberrant tumor protein p53 profile or tumours with high copy number alterations, NSMP/CN low = no specific molecular profile or tumours with low copy number alterations

Histological characteristics

Histological subtype	EC_Erlangen Scanner 1	EC_Erlangen Scanner 2	CPTAC-UCEC	TCGA-UCEC
Histological subtype absolute	289	287	120	450
Endometrioid all grades	289	287	120	342
(%)	100	100	100	76,0
Endometrioid grade 1	108	106	33	90
(%)	37,4	36,9	27,5	20,0
Endometrioid grade 2	118	118	58	108
(%)	40,8	41,1	48,3	24,0
Endometrioid grade 3 or high grade	63	63	28	144
(%)	21,8	22,0	23,0	32,0
Serous carcinoma	0	0	0	89
(%)				19,7
Clear-cell carcinoma	0	0	0	0
(%)				
Mixed serous and endometrioid carcinoma	0	0	0	19
(%)				4,2
Mixed serous and endometrioid carcinoma grade 2	0	0	0	18
(%)				
Mixed serous and endometrioid carcinoma grade 3	0	0	0	1
(%)				
Other	0	0	0	0

Supplementary Table 3: Histological stratification. The table shows the histological stratification of included cases sorted by cohort. EC_Erlangen Scanner 1 = WSIs scanned by scanner P1000, EC_Erlangen Scanner 2 = whole slide images (WSI) scanned by scanner S210, TCGA-UCEC = The Cancer Genome Atlas Uterine Corpus Endometrial Carcinoma Collection, CPTAC-UCEC = Clinical Proteomic Tumor Analysis Consortium Uterine Corpus Endometrial Carcinoma Collection.

Supplementary Results

Supplementary Results for benchmarking of feature extractors, normalization method and scanning hardware

Benchmarking on external testing data

Configuration	Subtype	Mean AUROC	std	95% CI
CTP Scanner 1+2, with stain normalization	*POLE*mut	0,568	0,090	0,504-0,632
	MMRd_MSI	0,619	0,032	0,596-0,642
	p53abn	0,802	0,025	0,784-0,820
	NSMP	0,567	0,043	0,536-0,598
	non-tumor	0,947	0,019	0,933-0,961
	macro-average tumor subtypes	0,639		
	macro-average	0,701		
CTP Scanner 1, with stain normalization	*POLE*mut	0,576	0,070	0,526-0,626
	MMRd_MSI	0,597	0,046	0,0564-0,630
	p53abn	0,806	0,021	0,791-0,821
	NSMP	0,576	0,037	0,550-0,602
	non-tumor	0,945	0,033	0,921-0,969
	macro-average tumor subtypes	0,639		
	macro-average	0,700		
CTP Scanner 2, with stain normalization	*POLE*mut	0,572	0,081	0,514-0,630
	MMRd_MSI	0,592	0,058	0,551-0,633
	p53abn	0,812	0,023	0,796-0,828
	NSMP	0,592	0,054	0,553-0,631
	non-tumor	0,930	0,049	0,895-0,965
	macro-average tumor subtypes	0,642		
	macro-average	0.700		
UNI Scanner 1+2, with stain normalization	*POLE*mut	0,639	0,059	0,597-0,681
	MMRd_MSI	0,660	0,039	0,632-0,688
	p53abn	0,827	0,040	0,798-0,856
	NSMP	0,624	0,032	0,601-0,647
	non-tumor	0,955	0,016	0,944-0,966
	macro-average tumor subtypes	0,688		
	macro-average	0,741		
UNI Scanner 1, with stain normalization	*POLE*mut	0,627	0,066	0,580-0,674
	MMRd_MSI	0,650	0,036	0,624-0,676
	p53abn	0,830	0,025	0,812-0,848
	NSMP	0,622	0,023	0,606-0,638

	non-tumor	0,951	0,014	0,941-0,961
	macro-average tumor subtypes	0,682		
	macro-average	0,736		
UNI Scanner 2, with stain normalization	*POLE*mut	0,645	0,066	0,598-0,692
	MMRd_MSI	0,671	0,020	0,657-0,685
	p53abn	0,828	0,033	0,804-0,852
	NSMP	0,631	0,021	0,616-0,646
	non-tumor	0,945	0,014	0,935-0,955
	macro-average tumor subtypes	0,694		
	macro-average	0,744		
CTP Scanner 1+2, no stain normalization	*POLE*mut	0,647	0,098	0,577-0,717
	MMRd_MSI	0,661	0,220	0,645-0,677
	p53abn	0,834	0,025	0,816-0,852
	NSMP	0,661	0,036	0,635-0,687
	non-tumor	0,964	0,037	0,938-0,990
	macro-average tumor subtypes	0,701		
	macro-average	0,753		
CTP Scanner 1, no stain normalization	*POLE*mut	0,633	0,068	0,584-0,682
	MMRd_MSI	0,660	0,028	0,640-0,680
	p53abn	0,833	0,024	0,816-0,850
	NSMP	0,651	0,027	0,632-0,670
	non-tumor	0,965	0,057	0,924-1,006
	macro-average tumor subtypes	0,694		
	average	0,748		
CTP Scanner 2, no stain normalization	*POLE*mut	0,660	0,082	0,601-0,719
	MMRd_MSI	0,660	0,040	0,631-0,689
	p53abn	0,832	0,028	0,812-0,852
	NSMP	0,661	0,024	0,644-0,678
	non-tumor	0,967	0,033	0,943-0,991
	macro-average tumor subtypes	0,703		
	macro-average	0,756		
UNI Scanner 1+2, no stain normalization	*POLE*mut	0,645	0,084	0,585-0,705
	MMRd_MSI	0,701	0,020	0,687-0,715
	p53abn	0,846	0,026	0,827-0,865
	NSMP	0,681	0,028	0,661-0,701
	non-tumor	0,932	0,018	0,919-0,945
	macro-average tumor subtypes	0,718		
	macro-average	0,761		
	*POLE*mut	0,624	0,052	0,587-0,661

UNI Scanner 1, no stain normalization	MMRd_MSI	0,686	0,029	0,665-0,707
	p53abn	0,850	0,024	0,833-0,867
	NSMP	0,655	0,031	0,633-0,677
	non-tumor	0,940	0,020	0,926-0,954
	macro-average tumor subtypes	0,704		
	macro-average	0,751		
UNI Scanner 2, no stain normalization	*POLE*mut	0,633	0,057	0,592-0,674
	MMRd_MSI	0,694	0,023	0,678-0,710
	p53abn	0,841	0,020	0,827-0,855
	NSMP	0,668	0,023	0,652-0,684
	non-tumor	0,941	0,023	0,925-0,957
	macro-average tumor subtypes	0,709		
	macro-average	0,755		

Supplementary Table 4: Benchmarking AUROC scores on external testing data. Mean class-wise area under the receiver operating characteristic curve (AUROC), standard deviation and 95% confidence interval (CI) for the independent test cohort from Erlangen (n=289) deployed on the ten cross-validated models among all configurations. We performed 10-fold cross-validation to train ten models on TCGA-UCEC and CPTAC-UCEC, which were then used for the subsequent external testing. All reported results were measured for external testing on our independent real-world cohort (n=289). CTP = Feature Extraction with CTransPath, UNI = Feature Extraction with UNI, Scanner 1+2 = image data from both scanners, Scanner 1 = Scanner P1000, Scanner 2 = Scanner S210, *POLE*mut = DNA polymerase epsilon ultra-mutated subtype, MMRd_MSI = mismatch repair protein deficiency or microsatellite instability, p53abn = aberrant tumor protein p53 profile, NSMP = no specific molecular profile.

**Configuration**	**Subtype**	**Precision mean**	**Precision std**	**Recall mean**	**Recall std**	**F1-Score mean**	**F1-Score std**
CTP Scanner 1+2, with stain normalization	MMRd_MSI	0,319	0,116	0,387	0,246	0,324	0,14
	NSMP	0,67	0,064	0,448	0,23	0,499	0,174
	*POLE*mut	0,075	0,032	0,219	0,157	0,102	0,038
	non-tumor	0,023	0,024	0,6	0,516	0,045	0,045
	p53abn	0,333	0,422	0,1	0,161	0,103	0,126
CTP Scanner 1, with stain normalization	MMRd_MSI	0,316	0,136	0,476	0,368	0,308	0,155
	NSMP	0,627	0,229	0,319	0,244	0,383	0,215
	*POLE*mut	0,037	0,034	0,269	0,321	0,061	0,059
	non-tumor	0,004	0,011	0,1	0,316	0,007	0,022
	p53abn	0,26	0,363	0,056	0,094	0,082	0,129
CTP Scanner 2, with stain normalization	MMRd_MSI	0,279	0,115	0,395	0,259	0,299	0,14
	NSMP	0,697	0,068	0,38	0,19	0,463	0,176
	*POLE*mut	0,153	0,3	0,312	0,299	0,094	0,055
	non-tumor	0,014	0,025	0,3	0,483	0,026	0,047
	p53abn	0,432	0,415	0,078	0,079	0,125	0,12

UNI Scanner 1+2, with stain normalization	MMRd_MSI	0,487	0,131	0,261	0,188	0,286	0,135
	NSMP	0,724	0,062	0,431	0,285	0,474	0,263
	*POLE*mut	0,092	0,037	0,556	0,205	0,152	0,048
	non-tumor	0,024	0,043	0,3	0,483	0,044	0,078
	p53abn	0,675	0,25	0,261	0,183	0,32	0,142
UNI Scanner 1, with stain normalization	MMRd_MSI	0,384	0,069	0,261	0,22	0,26	0,147
	NSMP	0,74	0,096	0,325	0,197	0,411	0,203
	*POLE*mut	0,091	0,042	0,681	0,227	0,153	0,05
	non-tumor	0	0	0	0	0	0
	p53abn	0,711	0,246	0,233	0,187	0,281	0,124
UNI Scanner 2, with stain normalization	MMRd_MSI	0,497	0,219	0,34	0,249	0,318	0,143
	NSMP	0,723	0,053	0,424	0,227	0,494	0,197
	*POLE*mut	0,093	0,03	0,506	0,225	0,15	0,039
	non-tumor	0,008	0,026	0,1	0,316	0,015	0,049
	p53abn	0,626	0,26	0,306	0,092	0,379	0,086
CTP Scanner 1+2, no stain normalization	MMRd_MSI	0,31	0,18	0,109	0,11	0,15	0,131
	NSMP	0,676	0,02	0,753	0,113	0,708	0,053
	*POLE*mut	0,109	0,063	0,262	0,141	0,131	0,051
	non-tumor	0,047	0,064	0,4	0,516	0,083	0,112
	p53abn	0,486	0,239	0,278	0,199	0,284	0,099
CTP Scanner 1, no stain normalization	MMRd_MSI	0,495	0,187	0,238	0,173	0,276	0,131
	NSMP	0,684	0,033	0,679	0,112	0,676	0,058
	*POLE*mut	0,09	0,047	0,262	0,164	0,127	0,063
	non-tumor	0,14	0,307	0,5	0,527	0,172	0,306
	p53abn	0,46	0,089	0,278	0,101	0,334	0,07
CTP Scanner 2, no stain normalization	MMRd_MSI	0,383	0,184	0,182	0,187	0,21	0,17
	NSMP	0,714	0,051	0,64	0,177	0,656	0,092
	*POLE*mut	0,089	0,033	0,381	0,225	0,134	0,046
	non-tumor	0,083	0,104	0,5	0,527	0,138	0,168
	p53abn	0,453	0,218	0,261	0,176	0,274	0,102
UNI Scanner 1+2, no stain normalization	MMRd_MSI	0,471	0,084	0,463	0,138	0,445	0,043
	NSMP	0,726	0,032	0,66	0,157	0,681	0,073
	*POLE*mut	0,164	0,132	0,169	0,125	0,148	0,1
	non-tumor	0	0	0	0	0	0
	p53abn	0,541	0,282	0,378	0,141	0,384	0,062
UNI Scanner 1, no stain normalization	MMRd_MSI	0,487	0,111	0,351	0,195	0,369	0,135
	NSMP	0,718	0,031	0,563	0,174	0,615	0,109
	*POLE*mut	0,096	0,054	0,362	0,228	0,137	0,065
	non-tumor	0,006	0,019	0,1	0,316	0,011	0,035
	p53abn	0,592	0,265	0,406	0,196	0,409	0,103
UNI Scanner 2, no stain normalization	MMRd_MSI	0,479	0,079	0,281	0,151	0,324	0,125
	NSMP	0,702	0,032	0,689	0,185	0,679	0,097
	*POLE*mut	0,142	0,106	0,269	0,156	0,148	0,046

	non-tumor	0,011	0,025	0,2	0,422	0,02	0,047
	p53abn	0,554	0,187	0,372	0,123	0,419	0,092

Supplementary Table 5: Benchmarking additional metrics on external testing data. Mean class-wise precision score, recall and F1-score with standard deviation for the independent test cohort from Erlangen (n=289) deployed on the ten cross-validated models among all configurations.. We performed 10-fold cross-validation to train ten models on TCGA-UCEC and CPTAC-UCEC, which were then used for the subsequent external testing. All reported results were measured for external testing on our independent real-world cohort (n=289). CTP = Feature Extraction with CTransPath, UNI = Feature Extraction with UNI, Scanner 1+2 = image data from both scanners, Scanner 1 = Scanner P1000, Scanner 2 = Scanner S210, *POLE*mut = DNA polymerase epsilon ultra-mutated subtype, MMRd_MSI = mismatch repair protein deficiency or microsatellite instability, p53abn = aberrant tumor protein p53 profile, NSMP = no specific molecular profile.

Supplementary results for the configuration UNI, without stain normalization and the use of image data from both scanners

Model performance on validation data

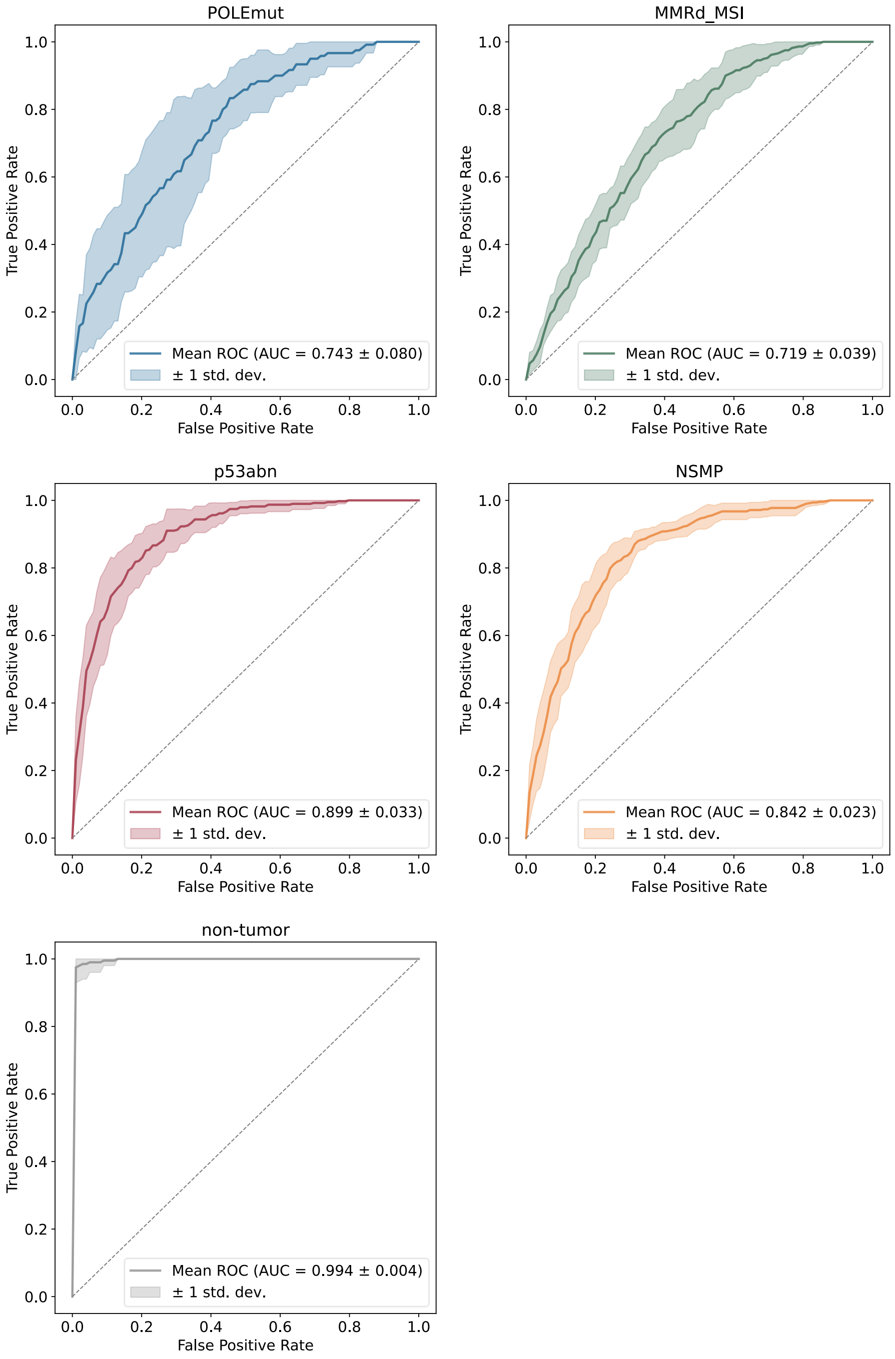

Supplementary Figure 2: Model performance on validation data. We performed 10-fold cross-validation to train ten models on TCGA-UCEC and CPTAC-UCEC. Mean one-vs-all classwise area under the receiver operating characteristic curves (AUROC) were obtained from ten different

data splits among training and validation datasets. Feature extraction was performed with UNI without stain normalization, using image data from both scanners. *POLE*mut = DNA polymerase epsilon ultra-mutated subtype, MMRd_MSI = mismatch repair protein deficiency or microsatellite instability, p53abn = aberrant tumor protein p53 profile, NSMP = no specific molecular profile.

FOLD	POLEmut	MMRd_MSI	p53abn	NSMP	non-tumor	mean
Fold-0	0,746	0,707	0,885	0,826	0,999	0,833
Fold-1	0,797	0,773	0,939	0,829	1,000	0,868
Fold-2	0,725	0,679	0,935	0,831	0,999	0,834
Fold-3	0,734	0,747	0,876	0,875	1,000	0,846
Fold-4	0,587	0,664	0,827	0,836	0,986	0,780
Fold-5	0,915	0,789	0,933	0,841	1,000	0,896
Fold-6	0,749	0,696	0,882	0,864	0,999	0,838
Fold-7	0,78	0,731	0,906	0,835	1,000	0,850
Fold-8	0,682	0,696	0,893	0,805	0,999	0,815
Fold-9	0,707	0,703	0,92	0,883	1,000	0,843

Supplementary Table 6: Model performance on validation data. We performed 10-fold cross-validation to train ten models on TCGA-UCEC and CPTAC-UCEC. Class-wise and mean one-vs-all classwise area under the receiver operating characteristic curves (AUROC) were obtained from ten different data splits among training and validation datasets. We selected a model (fold-1) with superior mean AUROCs on validation data for downstream analysis. Although the model of fold-5 exhibited higher mean AUROCs, we did not select that model with extreme validation performance for *POLE*mut exceeding two standard deviations above the mean to avoid overestimation. Feature extraction was performed with UNI without stain normalization, using image data from both scanners. *POLE*mut = DNA polymerase epsilon ultra-mutated subtype, MMRd_MSI = mismatch repair protein deficiency or microsatellite instability, p53abn = aberrant tumor protein p53 profile, NSMP = no specific molecular profile.

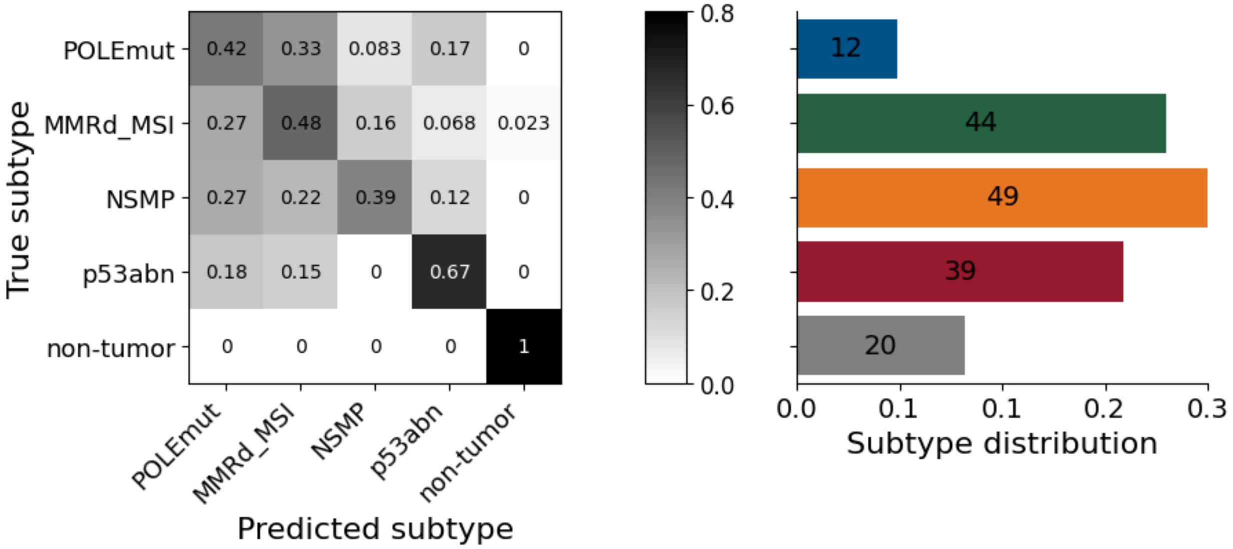

Supplementary Figure 3: Confusion matrix for fold-1 on the validation data. Normalized confusion matrix and distribution of molecular subtypes for the validation data (n=164). The confusion matrix demonstrates predominantly concordant predictions across all subtypes, supporting our selection of this model for single model evaluation. Feature extraction was performed with UNI without stain normalization, using image data from both scanners. *POLE*mut = DNA polymerase epsilon ultra-mutated subtype, MMRd_MSI = mismatch repair protein deficiency or microsatellite instability, p53abn = aberrant tumor protein p53 profile, NSMP = no specific molecular profile.

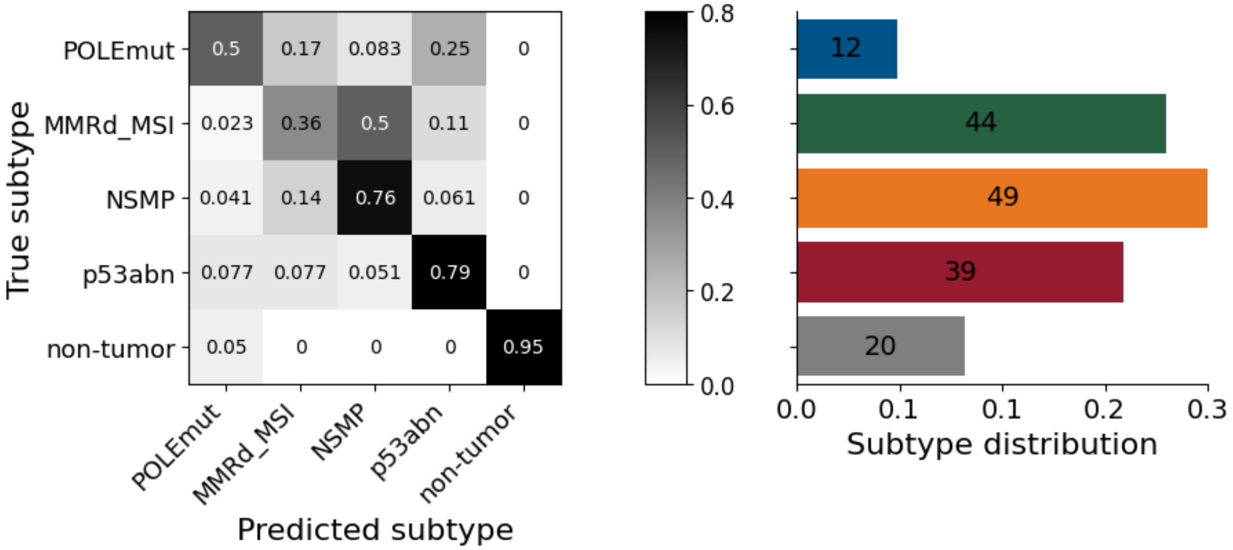

Supplementary Figure 4: Confusion matrix for fold-5 on the validation data. Normalized confusion matrix and distribution of molecular subtypes for the validation data (n=164). Feature extraction was performed with UNI without stain normalization, using image data from both scanners. *POLE*mut = DNA polymerase epsilon ultra-mutated subtype, MMRd_MSI = mismatch repair protein deficiency or microsatellite instability, p53abn = aberrant tumor protein p53 profile, NSMP = no specific molecular profile.

Model performance on external testing data

Fold	Subtype	AUROC	Precision	Recall	F1-score	Accuracy	n
fold-0	*POLE*mut	0,640	0,286	0,125	0,174	0,516	16
fold-0	MMRd_MSI	0,714	0,364	0,696	0,478	0,516	79
fold-0	p53abn	0,856	0,583	0,389	0,467	0,516	18
fold-0	NSMP	0,708	0,787	0,483	0,599	0,516	176
fold-1	*POLE*mut	0,766	0,214	0,375	0,273	0,561	16
fold-1	MMRd_MSI	0,715	0,449	0,557	0,497	0,561	79
fold-1	p53abn	0,862	0,333	0,389	0,359	0,561	18
fold-1	NSMP	0,682	0,745	0,597	0,662	0,561	176
fold-2	*POLE*mut	0,703	0,167	0,25	0,2	0,623	16
fold-2	MMRd_MSI	0,729	0,541	0,418	0,471	0,623	79
fold-2	p53abn	0,871	1	0,222	0,364	0,623	18
fold-2	NSMP	0,725	0,728	0,79	0,757	0,623	176
fold-3	*POLE*mut	0,580	0,069	0,125	0,089	0,516	16
fold-3	MMRd_MSI	0,708	0,402	0,595	0,48	0,516	79
fold-3	p53abn	0,865	0,333	0,222	0,267	0,516	18
fold-3	NSMP	0,664	0,738	0,545	0,627	0,516	176
fold-4	*POLE*mut	0,559	0,093	0,312	0,143	0,478	16
fold-4	MMRd_MSI	0,669	0,441	0,38	0,408	0,478	79
fold-4	p53abn	0,808	0,333	0,611	0,431	0,478	18
fold-4	NSMP	0,661	0,697	0,523	0,597	0,478	176
fold-5	*POLE*mut	0,780	0,333	0,125	0,182	0,664	16
fold-5	MMRd_MSI	0,696	0,519	0,342	0,412	0,664	79
fold-5	p53abn	0,870	1	0,222	0,364	0,664	18
fold-5	NSMP	0,715	0,71	0,903	0,795	0,664	176
fold-6	*POLE*mut	0,595	0,364	0,25	0,296	0,644	16
fold-6	MMRd_MSI	0,697	0,645	0,253	0,364	0,644	79
fold-6	p53abn	0,811	0,6	0,333	0,429	0,644	18
fold-6	NSMP	0,677	0,678	0,886	0,768	0,644	176
fold-7	*POLE*mut	0,682	0,118	0,125	0,121	0,578	16
fold-7	MMRd_MSI	0,713	0,519	0,354	0,421	0,578	79
fold-7	p53abn	0,863	0,667	0,333	0,444	0,578	18
fold-7	NSMP	0,661	0,693	0,744	0,718	0,578	176
fold-8	*POLE*mut	0,622	0	0	0	0,543	16
fold-8	MMRd_MSI	0,685	0,409	0,57	0,476	0,543	79
fold-8	p53abn	0,839	0,333	0,5	0,4	0,543	18
fold-8	NSMP	0,708	0,741	0,585	0,654	0,543	176
fold-9	*POLE*mut	0,535	0	0	0	0,495	16
fold-9	MMRd_MSI	0,672	0,425	0,468	0,446	0,495	79
fold-9	p53abn	0,810	0,222	0,556	0,317	0,495	18
fold-9	NSMP	0,637	0,738	0,545	0,627	0,495	176

Supplementary Table 7: Class-wise area under the receiver operating characteristic curve (AUROC), precision score, recall, F1-score and accuracy for model performance with UNI, without stain normalization and image data from both scanners. We performed 10-fold cross-validation to train ten models on TCGA-UCEC and CPTAC-UCEC, which were then used for the subsequent external testing. All reported results were measured for external testing on our independent real-world cohort (n=289). We selected fold-1 after validation to evaluate as single model for downstream analysis. *POLE*mut = DNA polymerase epsilon ultra-mutated subtype, MMRd_MSI = mismatch repair protein deficiency or microsatellite instability, p53abn = aberrant tumor protein p53 profile, NSMP = no specific molecular profile.

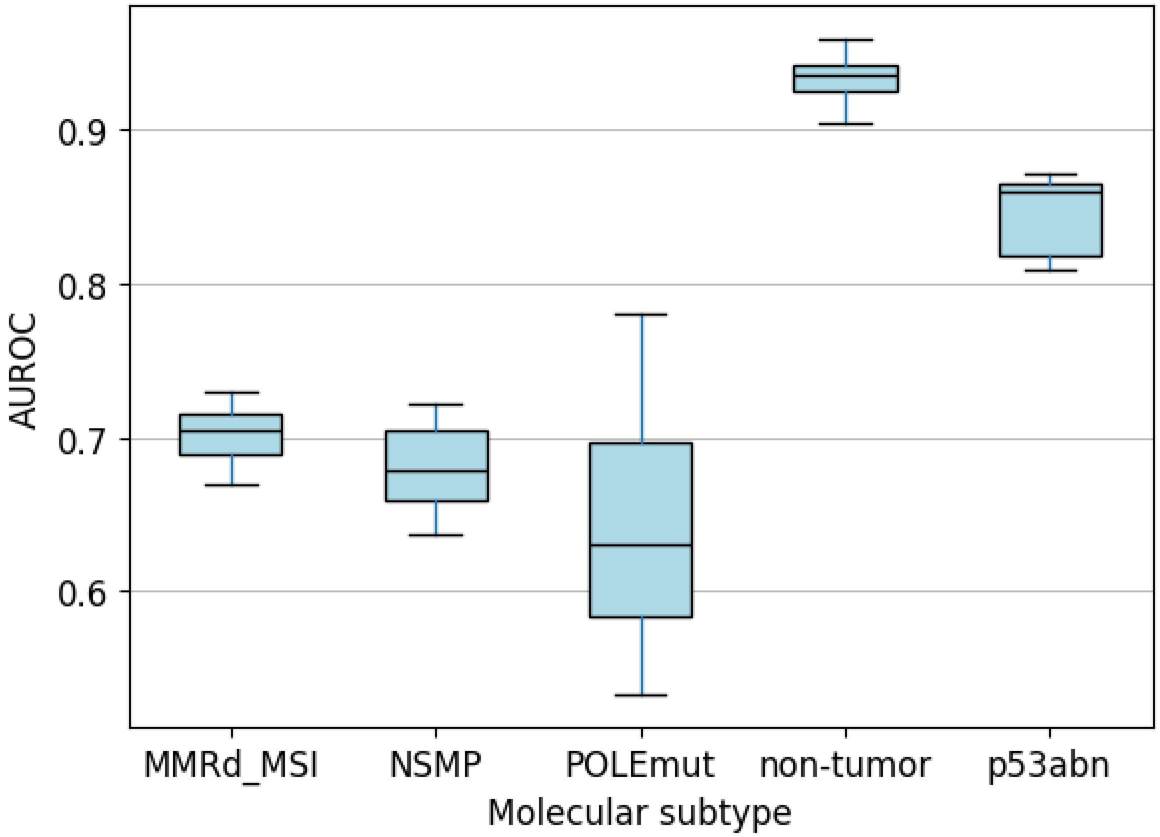

Supplementary Figure 5: Model performance with UNI, without stain normalization and image data from both scanners. Boxplot for the one-vs-all class-wise area under the receiver operating characteristic curve (AUROC) for the independent test cohort from Erlangen (n=289). The boxes visualize the quartiles of each distribution. The whiskers extend to the farthest data points within 1.5 times the interquartile range from the lower and upper quartiles. We performed 10-fold cross-validation to pretrain ten models on TCGA-UCEC and CPTAC-UCEC, which were then used for the subsequent external validation. All reported results were measured for external testing on our independent real-world cohort (n=289). *POLE*mut = DNA polymerase epsilon ultra-mutated subtype, MMRd_MSI = mismatch repair protein deficiency or microsatellite instability, p53abn = aberrant tumor protein p53 profile, NSMP = no specific molecular profile.

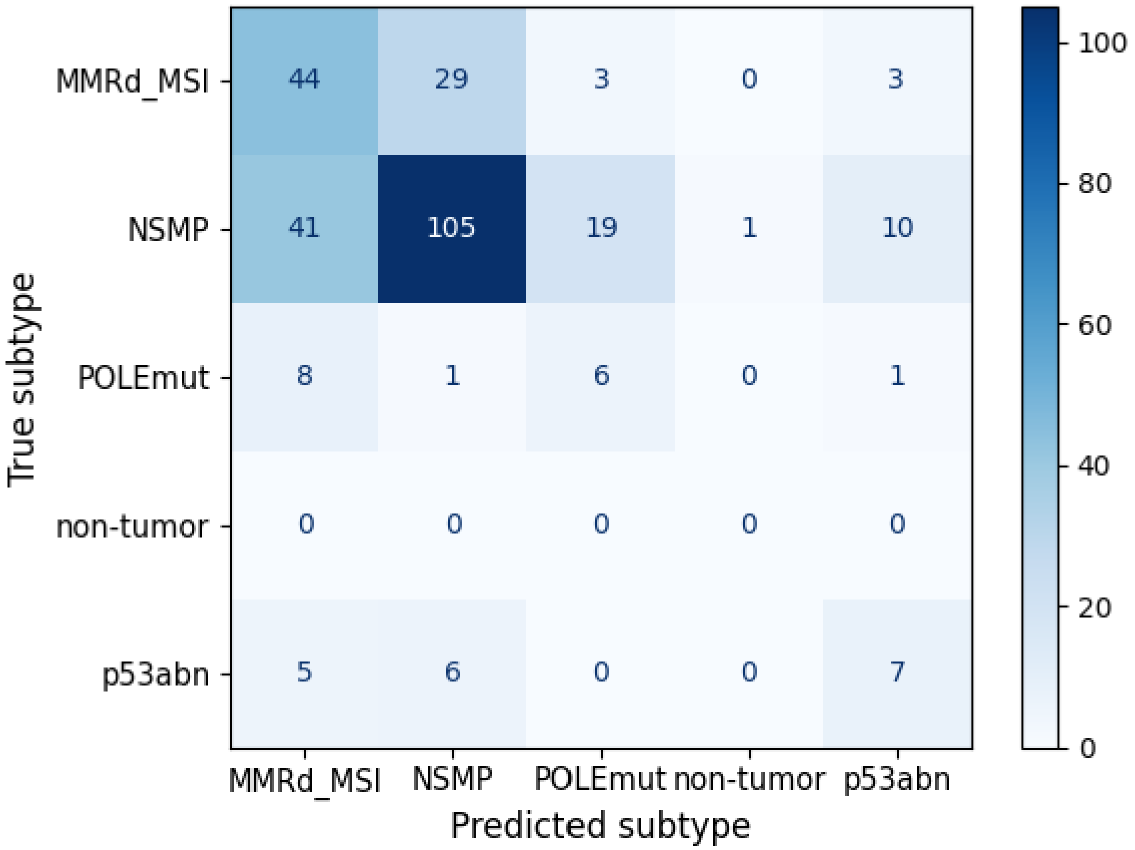

Supplementary Figure 6: Confusion matrix with absolute number of patients. All reported results were obtained from external testing on the Erlangen cohort (n=289) using the top-performing single model (fold-1). Feature extraction was performed with UNI without stain normalization, using image data from both scanners. *POLE*mut = DNA polymerase epsilon ultra-mutated subtype, MMRd_MSI = mismatch repair protein deficiency or microsatellite instability, p53abn = aberrant tumor protein p53 profile, NSMP = no specific molecular profile.

Supplementary results for single model performance stratified by grade

Concordant and non-concordant predictions stratified by grade

Molecular subtype	n	Grade	Concordant prediction	Non-concordant prediction	Non-concordant prediction distribution
*POLE*mut	16	All grades	6	10	
		Grade 1	2	2	1 MMRd_MSI<br>1 NSMP
		Grade 2	0	4	4 MMRd_MSI
		Grade 3	4	4	3 MMRd_MSI<br>1 p53abn
MMRd_MSI	79	All grades	44	35	
		Grade 1	4	15	2 *POLE*mut<br>1 p53abn<br>12 NSMP
		Grade 2	25	15	2 p53abn<br>13 NSMP
		Grade 3	15	5	1 *POLE*mut<br>4 NSMP
p53abn	18	All grades	7	11	
		Grade 1	1	3	3 NSMP
		Grade 2	2	0	
		Grade 3	4	8	3 NSMP<br>5 MMRd_MSI
NSMP	176	All grades	105	71	
		Grade 1	58	22	9 MMRd_MSI<br>9 *POLE*mut
		Grade 2	44	29	4 p53abn<br>7 *POLE*mut<br>17 MMRd_MSI<br>1 non-tumor
		Grade 3	3	20	2 p53abn<br>15 MMRd_MSI<br>3 *POLE*mut
All patients	289	All grades	162	127	
	107	Grade 1	65	42	
	119	Grade 2	71	48	
	63	Grade 3	26	37	

Supplementary Table 8: Model predictions stratified by grade. The table shows the stratification of concordant and non-concordant predictions by grade. All reported results were obtained from external testing on the Erlangen cohort (n=289) using the top-performing single model. Feature

extraction was performed with UNI without stain normalization, using image data from both scanners. *POLE*mut = DNA polymerase epsilon ultra-mutated subtype, MMRd_MSI = mismatch repair protein deficiency or microsatellite instability, p53abn = aberrant tumor protein p53 profile, NSMP = no specific molecular profile.

AUROC and confusion matrices stratified by grade

Grade 1+2 – "low-grade": 226 cases, 136 concordant (60,2%), 90 non-concordant (39,8%)

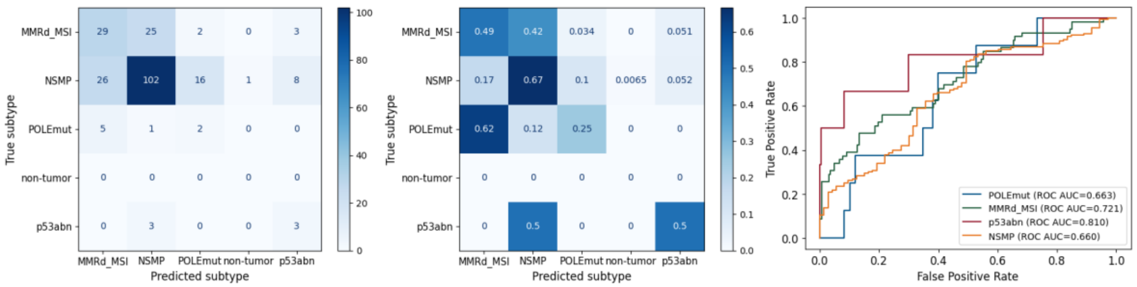

Supplementary Figure 7: Model performance for low-grade cases. Absolute and normalized confusion matrices and class-wise area under the receiver operating characteristic curve (ROC AUC) for the top-performing single model obtained from the low-grade group (n=226). All reported results were obtained from external testing on the Erlangen cohort using the top-performing single model. Feature extraction was performed with UNI without stain normalization, using image data from both scanners. *POLE*mut = DNA polymerase epsilon ultra-mutated subtype, MMRd_MSI = mismatch repair protein deficiency or microsatellite instability, p53abn = aberrant tumor protein p53 profile, NSMP = no specific molecular profile.

Grade 3 - "high-grade": 63 cases, 26 concordant (41,3%), 37 non-concordant (58,7%)

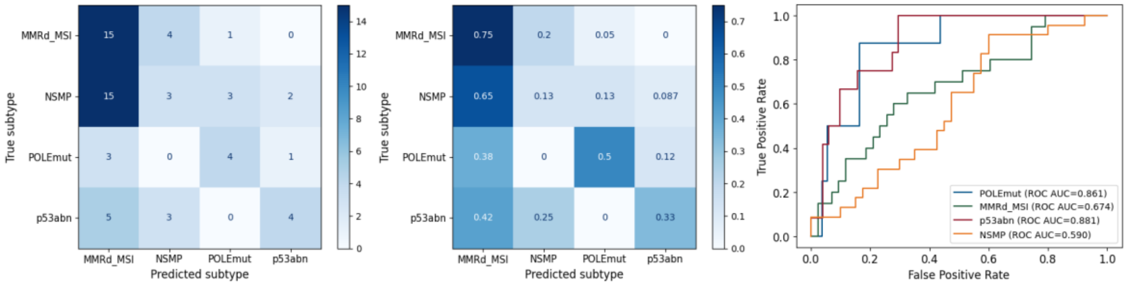

Supplementary Figure 8: Model performance for high-grade cases. Absolute and normalized confusion matrices and class-wise area under the receiver operating characteristic curve (ROC AUC) for the top-performing single model obtained from the high-grade group (n=63). All reported results were obtained from external testing on the Erlangen cohort using the top-performing single model. Feature extraction was performed with UNI without stain normalization, using image data from both scanners. *POLE*mut = DNA polymerase epsilon ultra-mutated subtype, MMRd_MSI = mismatch repair protein deficiency or microsatellite instability, p53abn = aberrant tumor protein p53 profile, NSMP = no specific molecular profile.

We tested the association between the predictions and the grade statistically with Fisher's exact test and accepted significance below a p-value of 0.05.

The prediction as morphoNSMP is significantly higher (p=1.7e-09) for low-grade cases than for high-grade cases (Table 7).

**Predicted subtype**	**Low-grade**	**High-grade**	
morphoNSMP	131	10	141
Not morphoNSMP	95	53	148
	226	63	289

Supplementary Table 9: Association morphoNSMP and low-grade. The table shows the association for the prediction as morphoNSMP and grade 1 and 2. morphoNSMP = classified as having no specific molecular profile by the model.

The prediction as morphoMMRd_MSI is significantly higher (p=1.7678e-06) for high-grade cases than for low grade cases (Table 8).

**Predicted subtype**	**Low-grade**	**High-grade**	
morphoMMRd_MSI	60	38	98
Not morphoMMRd_MSI	166	25	191
	226	63	289

Supplementary Table 10: Association morphoMMRd_MSI and high-grade. The table shows the association for the prediction as morphoMMRd_MSI and grade 3. morphoMMRd_MSI = classified as mismatch repair protein deficiency or microsatellite instability by the model.

There was no significant association between the morpho*POLE*mut or morphop53abn cases and the low-grade or high-grade characteristic.

AUROC and confusion matrices stratified by tumor cell count

Maximum of 10% tumor cell count: 48 cases

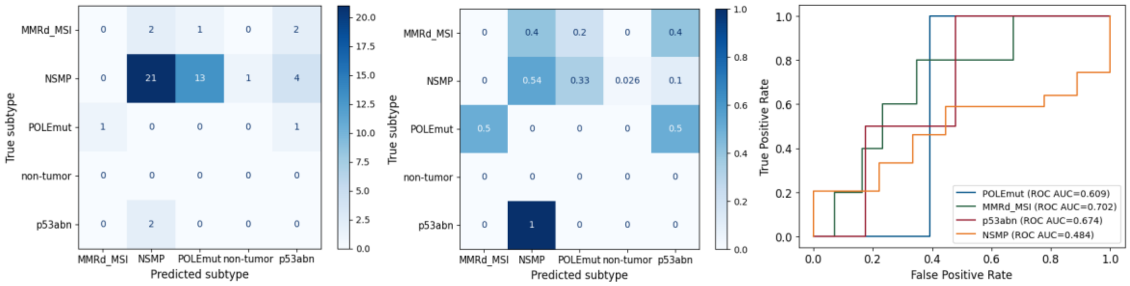

Supplementary Figure 9: Model performance for cases with low tumor cell count. Absolute and normalized confusion matrices and class-wise area under the receiver operating characteristic curve (ROC AUC) for the top-performing single model obtained from the subgroup with a maximum of 10% tumor cell count (n=48). All reported results were obtained from external testing on the Erlangen cohort using the top-performing single model. Feature extraction was performed with UNI without stain normalization, using image data from both scanners. *POLE*mut = DNA polymerase epsilon ultra-mutated subtype, MMRd_MSI = mismatch repair protein deficiency or microsatellite instability, p53abn = aberrant tumor protein p53 profile, NSMP = no specific molecular profile.

Report of multiple classifier cases

The Erlangen cohort included multiple classifier cases (n=4). There was one triple-classified case correctly classified as *POLE*mut. Two *POLE*mut-p53abn cases were falsely classified as MMRd_MSI. One *POLE*mut-MMRd_MSI case was falsely classified as MMRd_MSI.

Supplementary results for subtype specific slide-level scores

**Molecular subtype**	***POLE*mut**	**MMRd_MSI**	**p53abn**	**NSMP**
Number of patients	16	79	18	176
Mean score	0.307	0.442	0.370	0.441
Standard deviation	0.163	0.205	0.302	0.218
Minimum score	0.059	0.045	0.016	0.009
25%-Quartile	0.155	0.251	0.116	0.306
50%-Quartile	0.321	0.466	0.266	0.453
75%-Quartile	0.439	0.614	0.498	0.597
Maximum score	0.582	0.825	0.984	0.846

Supplementary Table 11: Subtype specific slide-level scores within the true molecular subtype. All reported results were obtained from external testing on the Erlangen cohort using the top-performing single model. Feature extraction was performed with UNI without stain normalization, using image data from both scanners. *POLE*mut = DNA polymerase epsilon ultra-mutated subtype, MMRd_MSI = mismatch repair protein deficiency or microsatellite instability, p53abn = aberrant tumor protein p53 profile, NSMP = no specific molecular profile.

Supplementary results for the explainability of morphological features

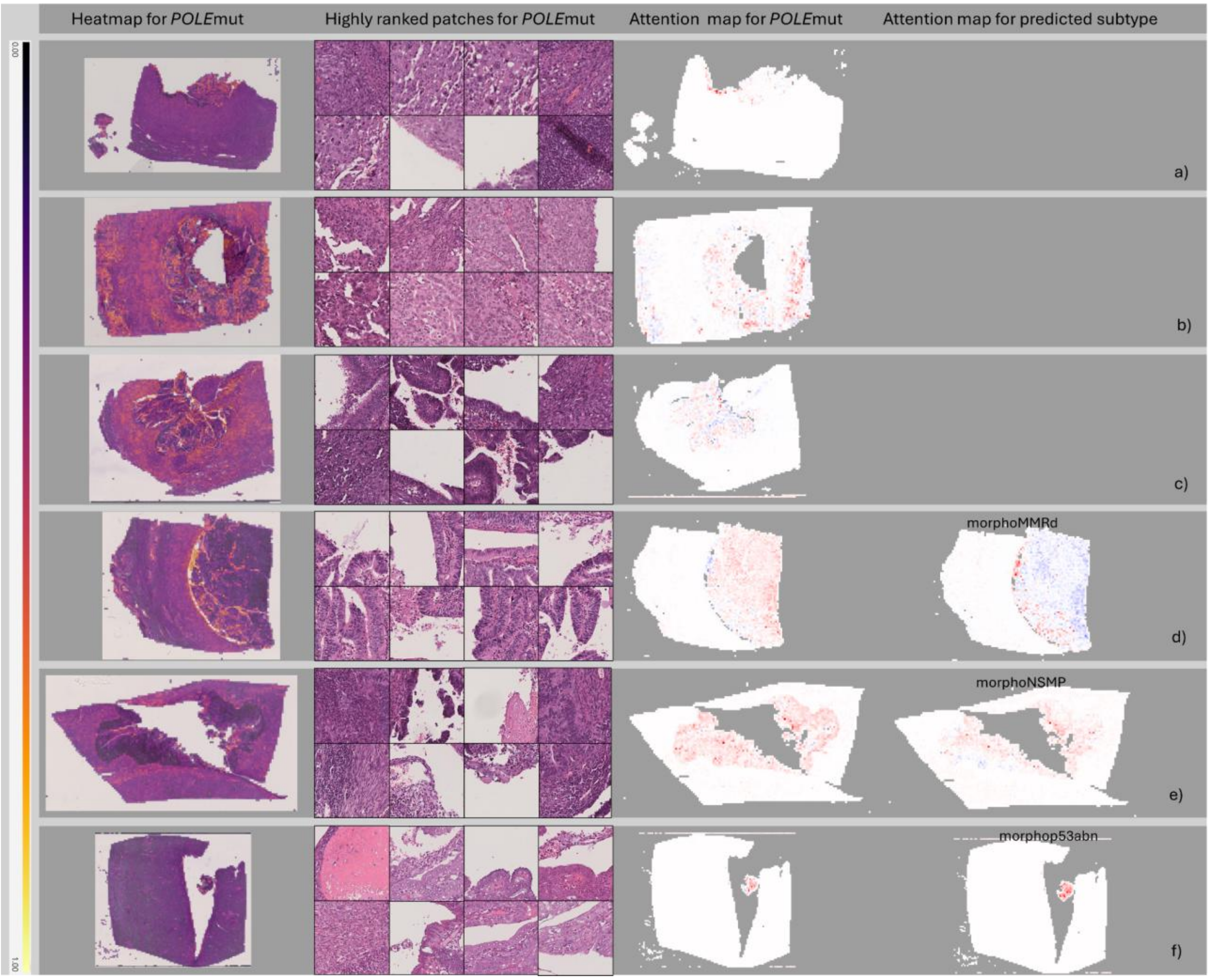

Supplementary Figure 10: Slide-level interpretability for representative *POLE*mut cases. Analysis of six cases of the DNA polymerase epsilon ultra-mutated subtype (*POLE*mut) molecular subtype with concordant and non-concordant predictions. Heatmaps were created with QuPath based on the tile-level scores for the *POLE*mut subtype (left), high scores in yellow, low scores in black with opacity set to 50%. The eight highest scored tiles were selected to characterize the subtype-specific morphology across varying slide-level scores (middle). Attention maps were generated with the Grad-CAM method, highlighting regions of high attention in red and low attention in blue (molecular subtype left, predicted subtype right). All slides were stained with haematoxylin and eosin (H&E). All underlying results were obtained from external testing on the Erlangen cohort using the top-performing single model. Feature extraction was performed with UNI without stain normalization, using image data from both scanners. morphoMMRd_MSI = classified as mismatch repair protein deficiency or microsatellite instability by the model, morphop53abn = classified as aberrant tumor protein p53 profile by the model, morphoNSMP = classified as having no specific molecular profile by the model. a) Grade 3, 15% tumor area; b) Grade 3, 30% tumor

area; c) Grade 1, 25% tumor area; d) Grade 2, 50% tumor area; e) Grade 1, 45% tumor area; f) Grade 3, 5% tumor area.

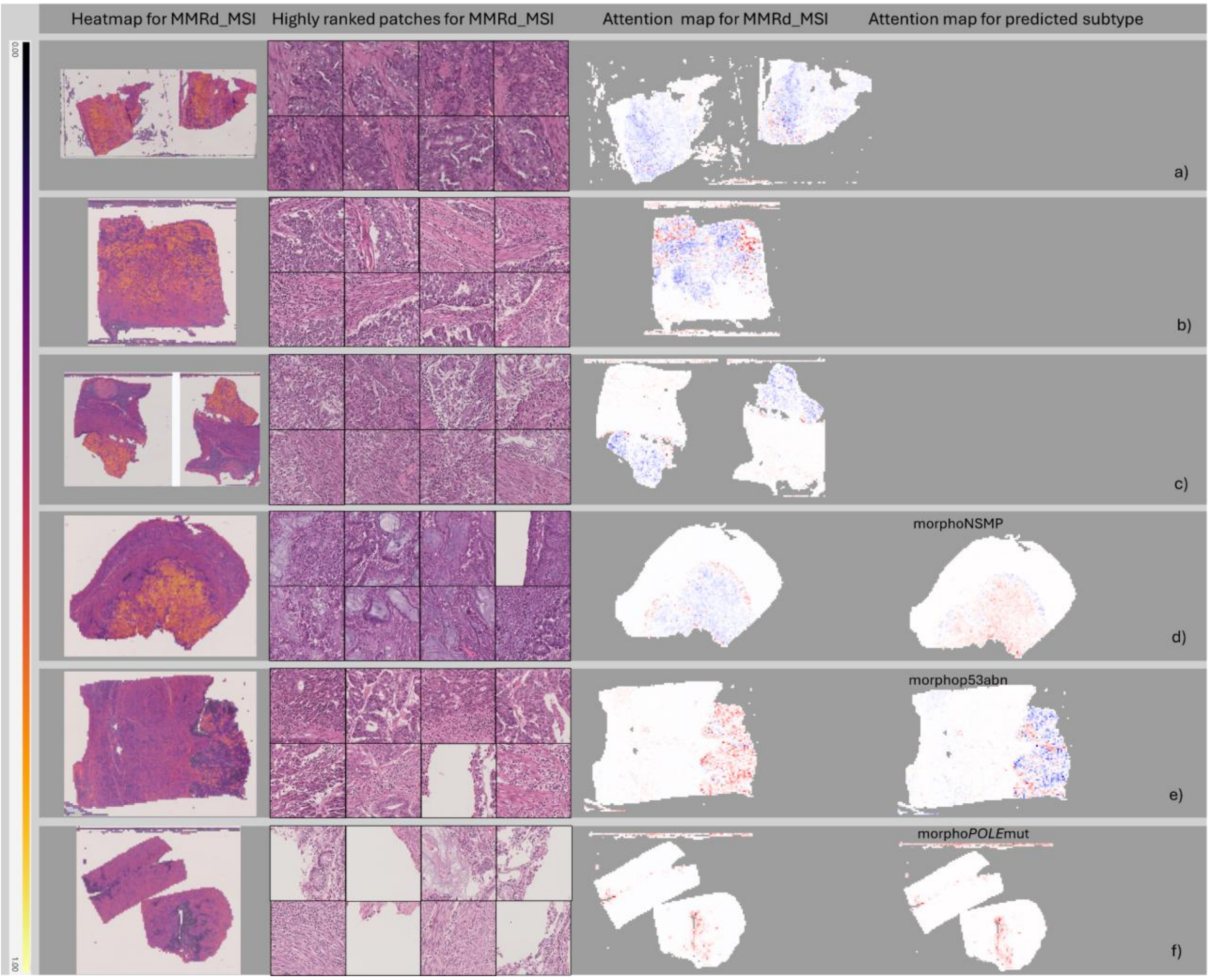

Supplementary Figure 11: Slide-level interpretability for representative MMRd_MSI cases. Analysis of six cases of the mismatch repair protein deficiency or microsatellite instability (MMRd_MSI) molecular subtype with concordant and non-concordant predictions. Heatmaps were created with QuPath based on the tile-level scores for the MMRd_MSI subtype (left), high scores in yellow, low scores in black with opacity set to 50%. The eight highest scored tiles were selected to characterize the subtype-specific morphology across varying slide-level scores (middle). Attention maps were generated with the Grad-CAM method, highlighting regions of high attention in red and low attention in blue (molecular subtype left, predicted subtype right). All slides were stained with haematoxylin and eosin (H&E). All underlying results were obtained from external testing on the Erlangen cohort using the top-performing single model. Feature extraction was performed with UNI without stain normalization, using image data from both scanners. morpho*POLE*mut = classified as DNA polymerase epsilon ultra-mutated subtype by the model, morphop53abn = classified as aberrant tumor protein p53 profile by the model, morphoNSMP = classified as having no specific molecular profile by the model. a) Grade 2, 25% Tumor area; b) Grade 3, 90% tumor area; c) Grade 2, 30% tumor area; d) Grade 2, 50% tumor area; e) Grade 2, 20% tumor area; f) Grade 1, 15% tumor area

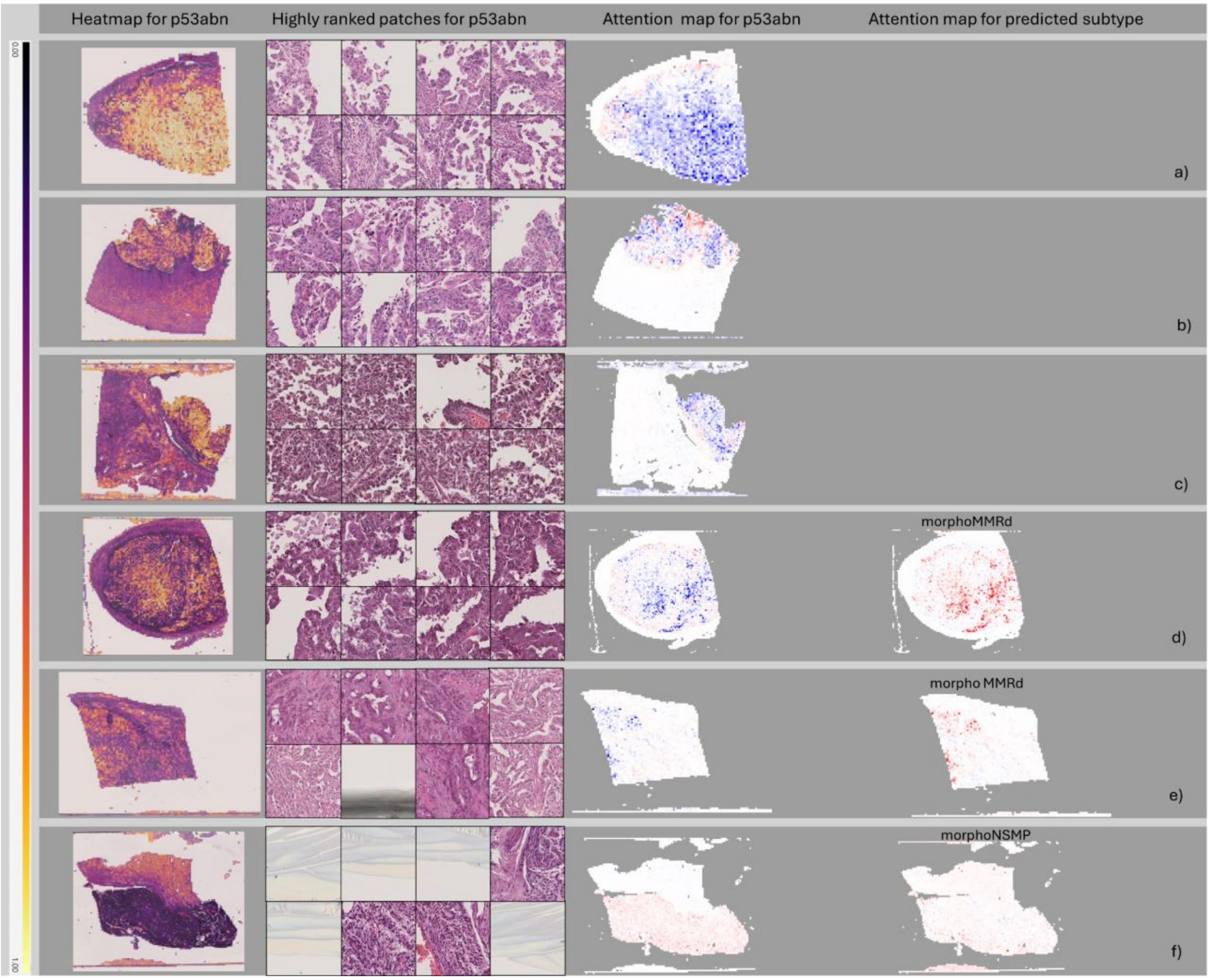

Supplementary Figure 12: Slide-level interpretability for representative p53abn cases. Analysis of six cases of the p53abn molecular subtype with concordant and non-concordant predictions. Heatmaps were created with QuPath based on the tile-level scores for the p53abn subtype (left), high scores in yellow, low scores in black with opacity set to 50%. The eight highest scored tiles were selected to characterize the subtype-specific morphology across varying slide-level scores (middle). Attention maps were generated with the Grad-CAM method, highlighting regions of high attention in red and low attention in blue (molecular subtype left, predicted subtype right). All slides were stained with haematoxylin and eosin (H&E). All underlying results were obtained from external testing on the Erlangen cohort using the top-performing single model. Feature extraction was performed with UNI without stain normalization, using image data from both scanners. morpho*POLE*mut = classified as DNA polymerase epsilon ultra-mutated subtype by the model, morphoMMRd_MSI = classified as mismatch repair protein deficiency or microsatellite instability by the model, morphop53abn = classified as aberrant tumor protein p53 profile by the model. a) Grade 2, 50% Tumor area; b) Grade 2, 40% tumor area; c) Grade 1, 35% tumor area; d) Grade 3, 75% tumor area; e) Grade 3, 25% tumor area; f) Grade 1, 55% tumor area

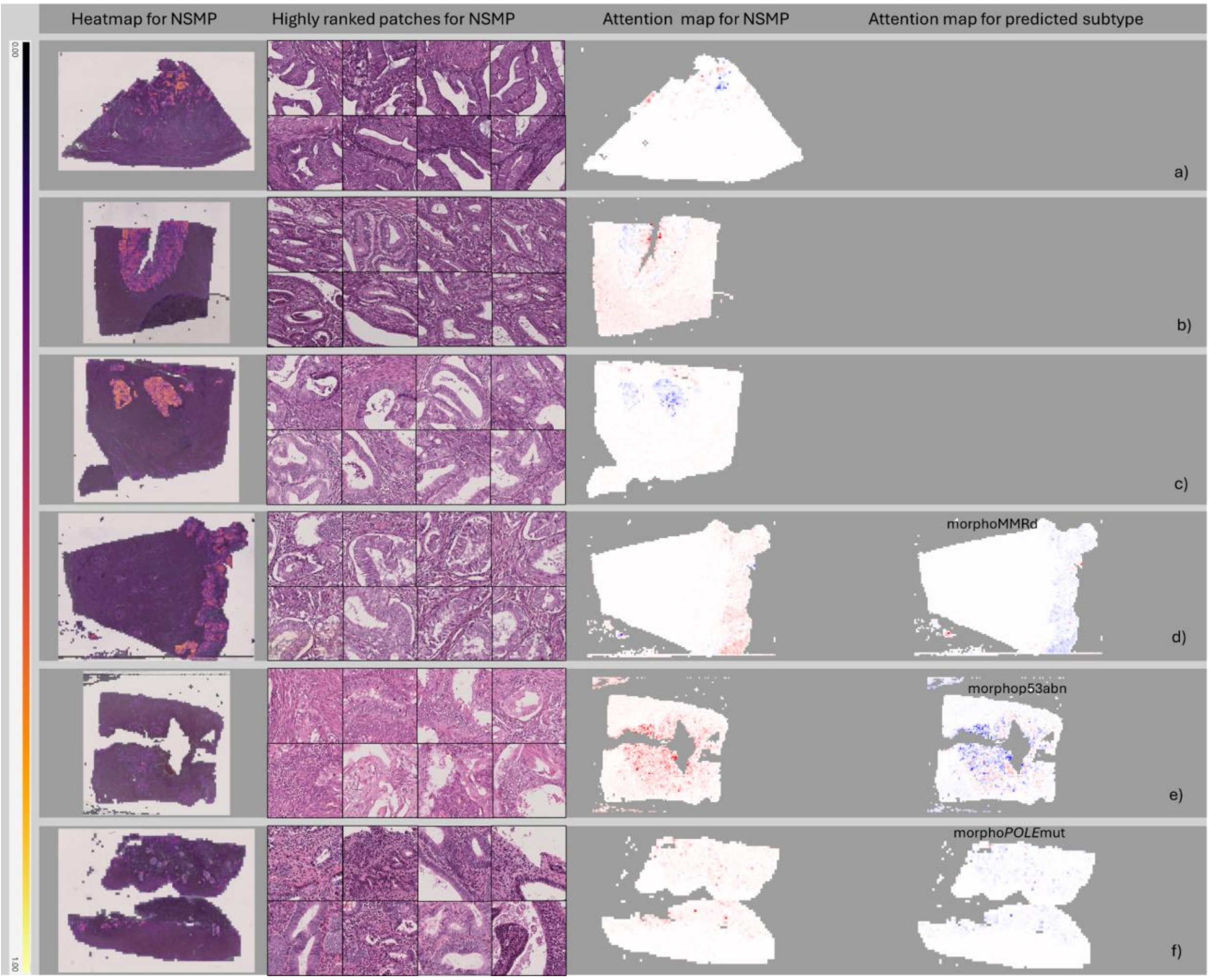

Supplementary Figure 13: Slide-level interpretability for representative NSMP cases. Analysis of six cases of the NSMP molecular subtype with concordant and non-concordant predictions. Heatmaps were created with QuPath based on the tile-level scores for the NSMP subtype (left), high scores in yellow, low scores in black with opacity set to 50%. The eight highest scored tiles were selected to characterize the subtype-specific morphology across varying slide-level scores (middle). Attention maps were generated with the Grad-CAM method, highlighting regions of high attention in red and low attention in blue (molecular subtype left, predicted subtype right). All slides were stained with haematoxylin and eosin (H&E). All underlying results were obtained from external testing on the Erlangen cohort using the top-performing single model. Feature extraction was performed with UNI without stain normalization, using image data from both scanners. morpho*POLE*mut = classified as DNA polymerase epsilon ultra-mutated subtype by the model, morphoMMRd_MSI = classified as mismatch repair protein deficiency or microsatellite instability by the model, morphoNSMP = classified as having no specific molecular profile by the model. a) Grade 1, 8% Tumor area; b) Grade 1, 30% tumor area, air bubble; c) Grade 1, 10% tumor area; d) Grade 2, 25% tumor area; e) Grade 3, 20% tumor area; f) Grade 1, 5% tumor area
